# Welander distal myopathy-associated TIA1 mutation exacerbates P-body and stress granule dynamics concomitant with nucleolar stress under oxidative stress

**DOI:** 10.1016/j.gendis.2025.101543

**Published:** 2025-01-23

**Authors:** Beatriz Ramos Velasco, José Alcalde, José M. Izquierdo

**Affiliations:** Center for Molecular Biology Severo Ochoa (CBM), Spanish National Research Council-Autonomous University of Madrid (CSIC/UAM), St/ Nicolás Cabrera 1, Madrid 28049, Spain

Welander distal myopathy (WDM) is a rare autosomal dominant inherited muscular dystrophy.^1^ WDM patients share an unusual haplotype on chromosome 2p13, where a heterozygous missense founder mutation (c.1362G > A; p.E384K; substitution of glutamic acid for lysine in the protein) has been identified in TIA1, an RNA-binding protein and a core component of stress granules (SGs).^2^ SGs are stress-induced non-membranous cytoplasmic aggregates from proteins and RNAs.^3^ The effects of the WDM-TIA1 mutation have been associated with abnormalities in the dynamics of TIA1-dependent SGs.^2^ Although its genetic cause is known, WDM is poorly understood. Understanding the WDM-associated molecular mechanisms, particularly the alterations in cellular homeostasis, is essential to identify therapeutic opportunities. Here, we sought to elucidate the molecular and cellular defects associated with mutant TIA1 expression, which may be partly responsible for the deficiencies observed in WDM patients.

We first explored the molecular differences of proteins and RNAs between TIA1^WT/WDM^-SGs under oxidative stress (Fig. 1A). We used isogenic HEK293 cell lines with inducible expression of GFP-tagged TIA1a^WT/WDM^ based on the Flp-In T-Rex system.^4^ We collected cells at 24 h post-induction and treated them with 0.5 mM NaAsO_2_ for 1 h. Cells were then fractionated by centrifugation and processed for immunopurification (Fig. 1A; Fig. S1A).^5^ The differential enrichment of several cellular components such as ectopic GFP-tagged TIA1^WT/WDM^, endogenous TIA1, U2AF2 (nuclear marker), and TUBA (cytoplasm marker) was examined (Figs. S1B–E). The fractions of enriched proteins and RNAs isolated with TRIzol reagent were used for mass spectrometry and RNA sequencing analysis, respectively (Fig. 1A). To uncover possible differences between TIA1^WT/WDM^-SGs, we used a label-free quantification mass spectrometry. Immunoprecipitated material was obtained from four independent experiments per condition, corresponding to the enriched TIA1-SG fractions under the indicated conditions of proteostasis and in the absence or presence of oxidative stress (Fig. 1B–E; Figs. S1D and E). The set of proteins differentially identified from the peptide precursors generated by trypsin digestion and their subsequent fractionation and spectrometric identification allowed the assignment of 183 identified proteins with a significance ≥ 20, according to the requirements established for a label-free quantification proteomic approach (Fig. 1B, C). The values represented are identified as red dots for positive values and green dots for negative values. Similarly, heat maps were generated for the tested conditions (Fig. 1B, C). The 183 proteins were translated to unique gene names (Fig. S2A). The functional nature and biological processes assigned to 84% of the 183 genes were RNA-binding proteins, as shown in the Venn diagrams with the EuRBPDB database (Figs. S2B and C). Notably, a significant number (32%) of the genes involved in the control of expression and post-transcriptional processing were found in those described in the core of G3BP1-dependent SGs identified by experimental immunoaffinity approaches similar to that employed in this study (Fig. S2D), with similar heterogeneity depending on the pairs compared and the experimental approaches used in the analysis of TIA1 and G3BP1 SG composition. The shared genes were clustered into biological process categories related to post-transcriptional regulatory aspects of gene expression control (Figs. S2E–H). Genes associated with TIA1-SGs with significant changes were also compared with those assigned to a P-body database and their functional categories of biological processes were analyzed (Figs. S3A–E). Collectively, we found a prevalent enrichment of RNA-binding proteins in TIA1-SGs clustered as P-bodies and canonical SG core-associated components.

**Figure 1:**
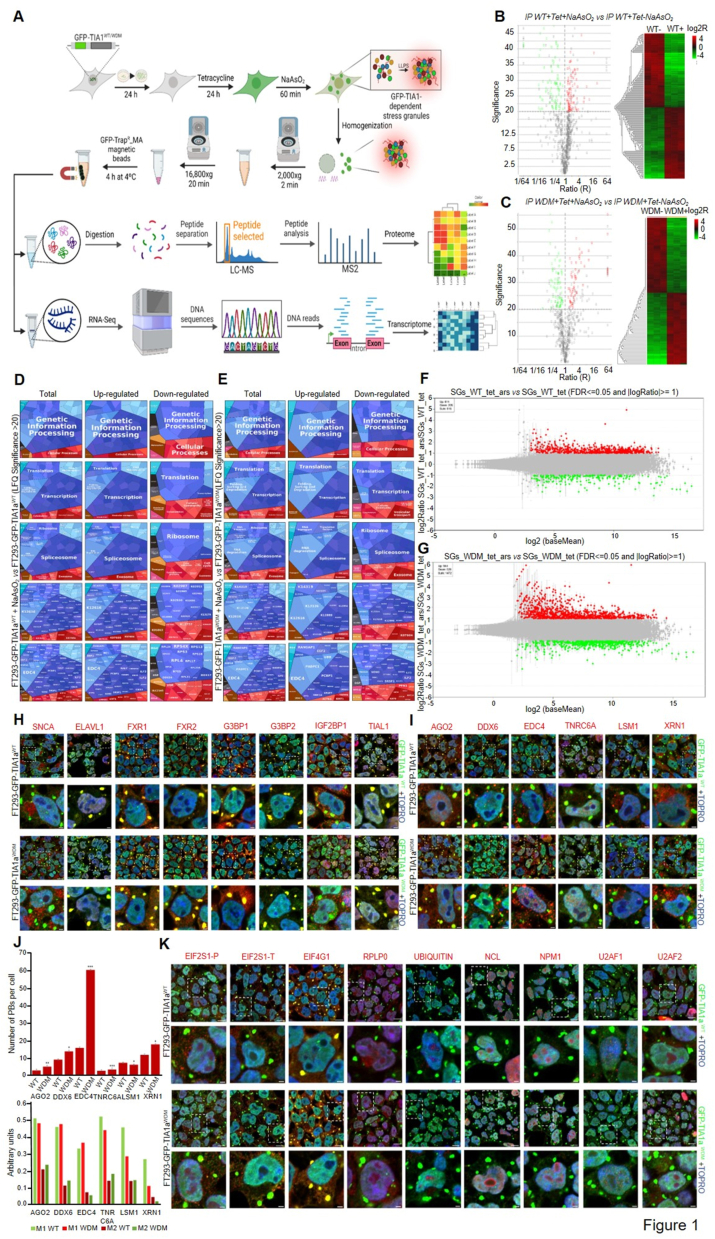
Differential transcriptomic and proteomic interactome of enriched wild-type (WT) and Welander distal myopathy (WDM) green fluorescent protein (GFP)-tagged TIA1-dependent stress granules (SGs) from genetically modified WT and WDM FT293-GFP-TIA1 cell lines under oxidative stress using affinity immunopurification, mass spectrometry, and RNA sequencing. **(A)** Details of workflow for the differential transcriptomic and proteomic analysis of WT and WDM-GFP-TIA1a-dependent SGs under oxidative stress. **(B**–**E)** Differential proteomic analysis of WT and WDM GFP-TIA1a-dependent SGs under oxidative stress. (B, C) Volcano and heat plots from proteomic analysis of immunoprecipitated WT (B) and WDM (C) TIA1-dependent SGs by label-free quantification proteomic analysis. (D, E) Proteomaps of the protein components (total, up-, and down-regulated proteins) identified in (D) and (E) by label-free quantification analysis with significance ≥20 in experimental conditions tested. **(F, G)** Transcriptomic profiling by RNA sequencing analysis of differentially immunoprecipitated genes in WT and WDM-GFP-TIA1a-dependent SGs under oxidative stress. M-versus-A plot representations of the distributions of over- (spots in red) and under- (spots in green) expressed genes (−1 > log fold change <1; false discovery rate <0.05) in WT (F) and WDM (G) FT293-GFP-TIA1a dependent SGs under oxidative stress. **(H)** Colocalization of selective components associated with GFP-TIA1a^WT^/^WDM^-dependent SGs under oxidative stress. Fluorescence images of GFP-TIA1a^WT^ and GFP-TIA1a^WDM^-expressing FT293 cells (green) immunolabeled for specific components (red) associated with TIA1-dependent SGs using specific antibodies against SNCA, ELAVL1, FXR1, FXR2, G3BP1, G3BP2, IGF2BP1, and TIAL1 proteins. Colocalization between components is shown in yellow. Nuclei were stained with To-Pro3 (blue in the merged image). Scale bars (image 60×, zoom 3 and zoom 3 plus crop) represent 10 μm and 2 μm, respectively. **(I)** Colocalization analysis of P-body specific components associated with GFP-TIA1a^WT^/^WDM^-dependent SGs under oxidative stress. Fluorescence images of GFP-TIA1a^WT^ and GFP-TIA1a^WDM^ FT293 cells (green) immunolabeled for specific components associated with P-bodies (red) and TIA1-dependent SGs using monoclonal antibodies against AGO2, DDX6, EDC4, TNRC6A, LSM1, and XRN1 proteins. Colocalization between components is shown in yellow. Nuclei were stained with To-Pro3 (blue in the merged image). Scale bars (image 60×, zoom 3 and zoom 3 plus crop) represent 10 μm and 2 μm, respectively. **(J)** Relative estimation of the number of P-bodies in WT and WDM FT293-GFP-TIA1a cells under oxidative conditions. Values represented are mean ± standard error of the mean (*n* = 32–44 cells) of the number of P-bodies per cell in each of the indicated markers. Significant differences (student’s *t*-test) are represented for the total number of SGs (∗*P* < 0.5, ∗∗*P* < 0.1, ∗∗∗*P* < 0.05) (upper graphic in J). The overlapping degree between signals was quantified using Manders' M1 and M2 colocalization coefficients. We can therefore interpret, in these simple cases, the M coefficients as the percentage of pixels in one channel that intersect with some signal in the other channel. M1 represents a fraction of A (TIA1-GFP-SGs) overlapping B (P-bodies), and M2 represents a fraction of B overlapping A. The represented values are mean ± standard error of the mean (*n* = 32–44 cells/3 fields) (lower graphic in J). **(K)** Colocalization analysis of translational machinery-specific components associated with GFP-TIA1a^WT^/^WDM^-dependent SGs under oxidative stress. Fluorescence images of GFP-TIA1a^WT^ and GFP-TIA1a^WDM^-expressing FT293 cells (green) immunolabeled for specific components associated with TIA1-dependent SGs (red) using specific antibodies against phosphorylated EIF2S1 (EIF2S1-P), total EIF2S1 (EIF2S1-T), EIF4G1, RPLP0, UBIQUITIN, NCL, NPM1, U2AF1, and U2AF2 proteins. Colocalization between components is shown in yellow. Nuclei were stained with To-Pro3 (blue in the merged image). Scale bars (image 60×, zoom 3 and zoom 3 plus crop) represent 10 μm and 2 μm, respectively. SNCA, synuclein alpha; ELAVL1, embryonic lethal abnormal vision, drosophila-like RNA binding protein 1; FXR1, fragile X mental retardation, autosomal protein-like protein 1; FXR2, FMR1 autosomal homolog 2; G3BP1, Ras-GTPase-activating protein SH3-domain-binding protein 1; G3BP2, Ras-GTPase-activating protein SH3-domain-binding protein 2; IGF2BP1, insulin-like growth factor 2 mRNA binding protein 1; TIAL1, TIA1-like protein. AGO2, argonaute RISC catalytic component 2; DDX6, DEAD-box helicase 6; EDC4, enhancer of mRNA decapping 4; TNRC6A, trinucleotide repeat containing adaptor 6A; LSM1, U6 SnRNA-associated Sm-like protein LSm; XRN1, 5′-3′ exoribonuclease 1; EIF2S1, eukaryotic translation initiation factor 2 subunit alpha; EIF4G1, eukaryotic translation initiation factor 4 gamma 1; RPLP0, ribosomal protein lateral stalk subunit P0; NCL, nucleolin; NPM1, nucleophosmin 1; U2AF1, U2 small nuclear RNA auxiliary factor 1; U2AF2, U2 small nuclear RNA auxiliary factor 2.

In the same vein, the clustering of proteins into functional categories and biological processes with positive or negative values for each of the WT and WDM experimental pairs was performed using the Proteomaps tool (Fig. 1D, E). The results suggested that the enriched categories of proteins identified were predominantly associated with regulatory aspects of RNA metabolism and post-transcriptional control of gene expression in the WT and WDM experimental situations analyzed. However, cellular processes related to DNA-dependent transcription and mRNA processing and splicing were slightly enhanced by GFP-TIA1a^WT^ expression, whereas cellular processes related to turnover, stability, and translation of mRNA were up-regulated and more interconnected with the expression of GFP-TIA1a^WDM^ (Fig. 1D, E). Overall, these observations indicate small regulatory nuances preferentially associated with the expression of the WDM mutant, which may suggest alterations in the dynamics of the corresponding cellular machineries responsible for turnover, stability, and/or mRNA translational efficiency in the context of WDM and the response to cellular oxidative stress.

We next used a massive sequencing approach (RNA sequencing) to identify RNAs whose expression was prevalently associated with TIA1^WT/WDM^-SG expression (Fig. 1F, G; Figs. S4–6). RNAs in SGs have been estimated to represent 75%–85% of the biological material. The RNAs identified from TIA1-SGs had a population distribution similar to that found in other SGs: 75% mRNAs, 10% long non-coding RNAs (lncRNAs), and the remaining processed RNAs of different origin (*e.g.*, pseudogene products, snRNAs, and snoRNAs). In addition, an enrichment in lncRNAs was observed in TIA1-SGs (Fig. S4). Furthermore, TIA1^WT/WDM^ immunoprecipitation resulted in a marked alteration in the expression under oxidative stress compared with that of equivalent cells without the stress stimulus. M-versus-A plots were used to illustrate these findings, showing up- and down-regulated genes for TIA1^WT^ (Fig. 1F) and TIA1^WDM^ (Fig. 1G). A total of 816 (of which 611 and 205 were up- and down-regulated, respectively) and 1472 (of which 944 and 528 were up- and down-regulated, respectively) RNAs were differentially expressed (false discovery rate < 0.05) in TIA1^WT^ and TIA1^WDM^ cells, respectively. To cluster differentially expressed genes and identify relevant biological processes associated with the ectopic expression of the RNA-binding proteins, transcripts were analyzed with the PANTHER tool. The top gene ontology (GO) categories were identified from the sum of significantly over- and under-expressed genes in TIA1^WT^ plus NaAsO_2_ versus TIA1^WT^ cells (Fig. S5). These categories recognized from differentially up-regulated RNAs involved processes related to development and membrane signaling dynamics (Fig. S5). By contrast, the top categories identified from differentially down-regulated genes were linked to important aspects of RNA metabolism and translation regulation. Also, the top GO categories from the sum of significantly over- and under-expressed genes in TIA1^WDM^ plus NaAsO_2_ versus TIA1^WDM^ cells were detected (Fig. S6). The top GO categories from differentially up-regulated RNAs were linked to specific aspects of neuronal and skeletal muscle morphogenesis and protein localizations (Fig. S6). However, the ones from differentially down-regulated genes were again connected to important aspects of cytoplasmic translation and SRP-dependent cotranslational machineries involving ribosomal protein mRNA processing.

We used immunofluorescence confocal microscopy to determine the subcellular localization of identified components related to TIA1-SGs (Fig. 1H), members of P-bodies (Fig. 1I), components of nucleolar and translational machineries (Fig. 1K), and other related and unrelated cellular components (Figs. S7–9). In many cases, we noted an overlap in the visualization of TIA1-SGs in cells with ectopic expression of WT or WDM TIA1 and the above subcellular components. Of note was the increase in the number and size of P-bodies and their preferential colocalization with TIA1^WDM^-SGs (Fig. 1I, J). These results suggest a concomitant and more robust change in the dynamics of P-bodies (an average 2-fold increase in EDC4 and DDX6 biomarkers together with more fragmentation of PBs) (Fig. S10) and TIA1-SGs associated with the expression of TIA1^WDM^ under oxidative stress conditions, which is in accord with the analysis of the transient proteomic and transcriptomic composition of TIA1^WT/WDM^-SGs in a nucleolar and ribosomal-dependent stress context. These findings also question us about the regulatory role of PB dynamics in post-transcriptional regulation associated with WDM-mutated TIA1 expression. In addition, we established the localization of components of the translational machinery as well as nucleolus and nucleus markers (Fig. 1K), suggesting a cell context in the nucleolar stress scenario on TIA1^WDM^ expression.

Collectively, these observations expand our knowledge of the WDM-mutation role at the interface of SGs and P-body dynamics, with targeted regulatory effects on the cell machinery of RNA decay/turnover and/or translation in a nucleolar stress-dependent scenario.

## Funding

This work was supported by grants RTI2018-098517B-I00 and PID2021-126152OB-I00 from 10.13039/501100008530MICIU/AEI/FEDER, UE, and a Fundación Ramón Areces institutional grant, Spain.

## CRediT authorship contribution statement

**Beatriz Ramos Velasco:** Validation, Software, Investigation, Formal analysis, Data curation. **José Alcalde:** Validation, Methodology, Investigation, Formal analysis. **José M. Izquierdo:** Writing – review & editing, Writing – original draft, Visualization, Supervision, Resources, Project administration, Methodology, Investigation, Funding acquisition, Formal analysis, Conceptualization.

## Conflict of interests

The authors declared no competing interests.
