# Supplementary material for: Welander distal myopathy-associated TIA1 mutation exacerbates P-body and stress granule dynamics concomitant with nucleolar stress under oxidative stress
Source: Genes Dis. 2025 Jan 23;12(6):101543. doi: 10.1016/j.gendis.2025.101543 (PMC12305575; doi:10.1016/j.gendis.2025.101543)
Supplement: Multimedia component 1 [file mmc1.pdf]

Supplementary data

Welander distal myopathy-associated TIA1 mutation exacerbates P-body and stress granule dynamics concomitant with nucleolar stress under oxidative stress

Beatriz Ramos Velasco, José Alcalde, and José M. Izquierdo*

Center for Molecular Biology Severo Ochoa (CBM)
Spanish National Research Council-
Autonomous University of Madrid (CSIC/UAM)
St/ Nicolás Cabrera 1
28049 Madrid
Spain

***Correspondence and proofs to:**
José M. Izquierdo
Email: jmizquierdo@cbm.csic.es

Materials and methods

Cell culture

The Flp-In^{TM} T-Rex^{TM}-HEK293 cell line (Invitrogen, Carlsbad, CA) and derivative FT293-GFP-TIA1a^{WT}and FT293-GFP-TIA1a^{WDM} cell lines were generated and maintained as described [1-4].

Immunofluoresecence confocal microscopy

For immunofluorescence analysis, cell cultures were grown on glass coverslips and fixed with 10% formalin (Merck, Darmstadt, Germany), as described [1-4]. We used the following primary antibodies: Ago2/EIF2C2 (rabbit polyclonal; 30735, Signalway Antibody, LLC, College Park, MD); SNCA (rabbit polyclonal, bs-0009R-TR, Bioss Antibodies Inc., Woburn, MA); ATG3 (rabbit polyclonal, PM034B); ATG7 (rabbit polyclonal, PM039B), ATG12 (mouse monoclonal, M154-3B), and ATG16L (rabbit polyclonal, PM040B) (all from Medical and Biological Laboratories LTD., Nagoya, Japan); β-actin (AC-74, Sigma, Madrid, Spain); DDX6 (rabbit polyclonal, 144-64249, RayBiotech, Norcross, GA); CNX (rabbit polyclonal, SPC-108B, StressMarq Inc., Victoria, Canada); EDC4 (rabbit polyclonal, bs-10953R, Bioss Antibodies; rabbit polyclonal, E-AB-66481, Elabscience); EIF2S1/P (rabbit polyclonal, 9721; Cell Signaling Technology, Danvers, MA), EIF2S1/T (mouse monoclonal, sc-133132; Santa Cruz Biotechnology, Santa Cruz, CA); FXR1 (rabbit polyclonal, DF12402, Affinity Bioreagents, Rockford, IL); FXR2 (rabbit polyclonal, OAAN01137, Aviva Systems Biology, San Diego, CA); G3BP1 (rabbit polyclonal, CSB-PA009116GA01HU, Cusabio Technology LLC, Houston, TX); G3BP2 (rabbit polyclonal, DF4387, Affinity Bioreagents); TNRC6A (rabbit polyclonal, DF13371, Affinity); hnRNPA1 (mouse monoclonal, gift from Dr Krainer's laboratory, CSHL); hnRNPA2/B1 (rabbit polyclonal, AF5272, Affinity Bioreagents); ELAVL1 (mouse monoclonal, sc-5261; Santa Cruz Biotechnology); p0 (gift from Dr Ballesta's laboratory, CBM); IGF2BP1 (rabbit polyclonal, bs-8683R, Bioss Antibodies); LAMP1 (mouse monoclonal, 1D4B, DSHB, Iowa City IA); LSM1 (rabbit polyclonal, A72475, EpiGentek, Farmingdale, NY); NCL (polyclonal rabbit; sc-22758; Abcam, Cambridge, UK); NPM1 (B23, sc-5564; Santa Cruz Biotechnology); NFE2L2 (rabbit polyclonal, bs-1074R-TR, Bioss Antibodies); PTBP1 (mouse monoclonal, Invitrogen); TIA1 (C-20, sc-1751; Santa Cruz Biotechnology); TIAL1 (C-18, sc-1749; Santa Cruz Biotechnology); TDP43 (rabbit polyclonal, gift from Dr Baralle's laboratory); TOM20 (mouse monoclonal, sc-17764, Santa Cruz

Biotechnology); α-tubulin (TUBA) (B-5-1-2, Sigma); U2AF1 (rabbit polyclonal, 60289-1-lg, Proteintech, Chicago, IL); U2AF2 (mouse monoclonal, gift from Dr Valcárcel's, Centre for Genomic Regulation (CRG)); XRN1 (rabbit polyclonal, DF2433, Affinity Bioreagents); VIM (mouse monoclonal, sc-32322, Santa Cruz Biotechnology). All primary antibodies were used at 1:100.

Subcellular fractionation and enrichment of stress granules

SGs were enriched from cells stably transfected with plasmids for GFP-TIA1a^{WT} and GFP-TIA1a^{WDM} expression, as described [5-8], with modifications. Cells were incubated with 0.5 mM $NaAsO_2$ for 1 h at 37ºC and snap-frozen on dishes. Cells were then scraped off, washed once with DMEM, snap-frozen with liquid nitrogen and stored at -70ºC. Cellular pellets were thawed on ice at 4ºC for 10 min and processed using lysis buffer (50 mM Tris HCl, pH 7.4, 100 mM KOAc, 2 mM MgOAc, 0.5 mM DTT, 50 μg/ml heparin, 0.5% NP-40, and a protease inhibitor cocktail) and passed through a 25G, 5/8" (0.5 mm × 16 mm) needle (BD Microlance 3, Becton Dickinson, Sunnyvale, CA). The lysate was centrifuged at 1,000 g for 5 min, and the supernatant was then centrifuged at 16,873 g for 20 min. The pellet containing SGs was resuspended in the lysis buffer and incubated with GFP-Trap magnetic agarose beads (Chromotek GmbH, Planegg-Martinsried, Germany) for 4 h. Beads were washed three times with washing buffer 1 (20 mM Tris HCl and 200 mM NaCl, pH 8.0), once with washing buffer 2 (20 mM Tris HCl and 500 mM NaCl, pH 8.0) and once with washing buffer 3 (lysis buffer containing 2 M urea). The proteins were eluted in 2× Laemmli sample buffer at 95ºC for 10 min. Total RNA was purified using TRIzol^{R} Reagent (Ambion, Life Technologies, Carlsbad, CA) [1-3].

Western blotting

Proteins were separated by 10% SDS-PAGE and transferred to a nylon membrane at 4ºC and 50 V for 2 h. The membrane was then blocked with 5% powdered milk in phosphate-buffer saline (pH 7.5) containing 0.1% Tween-20 (Sigma) (PBS/T). The membrane was then probed with specific antibodies against the indicated proteins. Membranes were incubated with primary antibodies in PBS/T containing 3% BSA (Sigma) overnight at 4ºC, washed, and then incubated with appropriate HRP-conjugated secondary antibodies at room temperature for 1 h. The ECL reagent (GE Healthcare, Madison, WI) for HRP was used as a developer. The antibodies used were the following: TIA1 (sc-1751 1/3,000), TIAL1 (sc-1749 1/3,000), and ELAVL1 (sc-5261 1/4,000) (all from Santa Cruz

Biotechnology); U2AF2 (generously provided by Prof. J. Valcárcel); and TUBA (T5168 1/5,000, Merck) [1-4]. Secondary antibodies were from goat, rabbit and mouse (Promega, Madison, WI), as appropriate.

In-Gel digestion

A pool of proteins from 4 independent biological samples was suspended in sample buffer (60 µl) and applied to 1.2-cm wide wells of a conventional SDS-PAGE gel (0.75 mm, 4% stacking, and 10% resolving). The run was stopped as soon as the front entered 3 mm into the resolving gel, such that the whole proteome was concentrated in the stacking/resolving gel interface. The unseparated protein bands were visualized by Coomassie staining (Coomassie Brilliant Blue G-250 reagent, Bio-Rad, Hercules, CA), excised, cut into slices (2 × 2 mm) and then placed into 0.5-ml microcentrifuge tubes [9]. The gel pieces were destained in acetonitrile:water (ACN:H_2O, 1:1) and then reduced and alkylated (disulfide bonds from cysteinyl residues were reduced with 10 mM DTT for 1 h at 56 ºC, and thiol groups were then alkylated with 10 mM iodoacetamide for 30 min at room temperature in the dark). Proteins were then digested *in situ* using sequencing grade trypsin (Promega), as described [10], with minor modifications. The gel pieces were shrunk by the removal of all liquids using sufficient ACN, which was then removed before the gel pieces were dried in a speedvac. The gel pieces were then re-swollen in 100 mM Tris-HCl pH 8, 10 mM $CaCl_2$ with 60 ng/µl trypsin at 5:1 protein:enzyme (w/w) ratio. The tubes were kept on ice for 2 h and then incubated at 37°C for 12 h. Digestion was stopped by the addition of 1% trifluoroacetic acid. Whole supernatants were dried down and then desalted onto ZipTip C18 Pipette tips (Millipore, Billercia, MA) or OMIX Pipette tips C18 (Agilent Technologies, Santa Clara, CA) for mass spectrometry analysis.

Reverse phase-liquid chromatography RP-LC-MS/MS analysis

The desalted protein digest was dried, resuspended in 10 µl 0.1% formic acid and analyzed by RP-LC-MS/MS in an Easy-nLC II system coupled to an ion trap LTQ-Orbitrap-Velos-Pro hybrid mass spectrometer (Thermo Scientific, Waltham, MA). The peptides were concentrated (on-line) by reverse phase chromatography using a 0.1 mm × 20 mm C18 RP precolumn (Thermo Scientific), and then separated using a 0.075 mm × 250 mm C18 RP column (Thermo Scientific) operating at 0.3 µl/min. Peptides were eluted using a 180-min dual gradient. The gradient profile was set as follows: 5−25% solvent B for 135 min, 25−40% solvent B for 45 min, 40−100% solvent B for 2 min and

100% solvent B for 18 min (Solvent A: 0.1% formic acid in water, solvent B: 0.1% formic acid, 80% ACN in water). ESI ionization was done using a stainless steel Nano-bore emitter, ID 30 μm (Proxeon Biosystems, Odense, Denmark), interface at 2.1 kV spray voltage with S-Lens of 60%. The Orbitrap resolution was set at 30.000 [11].

Peptides were detected in survey scans from 400 to 1600 amu (1 μscan), followed by twenty data-dependent MS/MS scans (Top 20), using an isolation width of 2 μ (in mass-to-charge ratio units), normalized collision energy of 35%, and dynamic exclusion applied during 60 second periods. Charge-state screening was enabled to reject unassigned and singly charged protonated ions [12].

Data processing

Peptide identification from raw data was carried out using PEAKS Studio X+ search engine (Bioinformatics Solutions Inc., Waterloo, Ontario, Canada). A database search was performed against Uniprot-*Homo sapiens* (75069 entries; UniProt release 06/2020) (decoy-fusion database). The following constraints were used for the searches: tryptic cleavage after Arg and Lys (semispecific), up to two missed cleavage sites, and tolerances of 20 ppm for precursor ions and 0.6 Da for MS/MS fragment ions, and the searches were performed allowing optional Met oxidation and Cys carbamidomethylation. The false discovery rate (FDR) for peptide spectrum matches (PSM) was limited to 0.01. Only those proteins with at least two unique peptides discovered from LC/MS/MS analyses were considered reliably identified [13-15].

The mass spectrometry proteomics data have been deposited in the ProteomeXchange Consortium *via* the PRIDE [16,17] partner repository with the dataset identifier PXD038028 and 10.6019/PXD038028. *Project Name: "Study of TIA1-dependent stress granules in homeostasis (WT) and Welander distal myopathy (WDM)". Project accession: PXD038028. Project DOI: 10.6019/PXD038028.*

Label-free quantitative data analysis

Peptide identification from raw data (technical triplicates) was carried out using the PEAKS Studio X+ search engine (Bioinformatics Solutions Inc.). The database search was performed against Uniprot-*Homo sapiens* (75069 entries; UniProt release 06/2020) (decoy-fusion database). The following constraints were used for the searches: tryptic cleavage after Arg and Lys (semispecific), up to two missed cleavage sites, and tolerances of 20 ppm for precursor ions and 0.6 Da for MS/MS fragment ions, and the searches were

performed allowing optional Met oxidation and Cys carbamidomethylation. FDRs for PSM was limited to 0.01. Only those proteins with at least two distinct peptides and at least one unique peptide discovered from LC/MS/MS analyses were considered reliably identified and used for quantification. Quantitation of peptides was performed with PEAKS Studio X+ search engine, selected “Label Free Quantification” under the “Quantifications” options using 20 ppm for mass error tolerance and 3 min for retention time shift tolerance. We used the total ion current (TIC) of the samples to calculate the normalization factors. Normalized abundance is calculated from the raw abundance divided by the normalization factor. The Quality (Cytosol: 12; IP: 15) and Avg. Intensity (Cytosol:2e5; IP:8e5) were used for Spectrum filter, and Significance (20, ANOVA) was used for peptide and protein abundance calculation. For protein quantification, we considered protein groups for peptide uniqueness, used only unique peptides for protein quantification, and modified peptides were excluded [18].

RNA isolation

Total RNA was isolated and purified using TRIzol^{R} Reagent (Ambion, Life Technologies) and the quality was checked on an Agilent 2100 Bioanalyzer (RIN >8) [3].

RNA-Seq analysis

Transcriptome sequencing (RNA-seq) was performed as strand-specific paired reads using Illumina technology. The RNA-seq approach consisted in the analysis of 10 different experimental conditions with three independent biological replicates for each experimental condition (30 samples in total). In this case, a massive sequencing kit was used after removal of ribosomal RNA with Ribo-Zero Gold nuclease (Epicentre). The specifications were as follows: "Truseq stranded total RNA with Ribo-Zero-Gold + NovaSeq6000 150PE (150 × 2 bp), 40 M total reads/sample (6 Gb/sample)". With the combination of 'Truseq' gen libraries (original from Illumina) and 'NovaSeq6000' sequencers, we achieved more than 90% read rate with Q30, according to Macrogen specifications. Subsequent analysis of the raw data files was performed at the Genomics and Proteomics Bioinformatics Service of the Centro Nacional de Biotecnología (CNB-CSIC) [3].

Data analysis

Quality check of paired-end short reads (in FASTQ format) was evaluated with FASTQC [19]. The presence of Illumina adapters was detected and reads were 3' trimmed, to a final

size of 100 nucleotides, with Trimmomatic [20]. Trimmed reads were aligned against the human genome using RNA-STAR [21] (--alignIntronMax 1000000). Optical and PCR duplicates were identified with 'MarkDuplicates' function of picard-tools/GATK suite [22]. Alignment files in SAM format were compressed, sorted and indexed with samtools [23]. Human genes were quantified using the featureCounts function from the Rsubread package [24] (strandSpecific=2, primaryOnly=TRUE, ignoreDup=TRUE). Alignments were visualized with the IGV broswer [25]. Differential expression values and their statistical significance were calculated with DESeq2 [26] using the lfcShrink function (type='apeglm') [27]. Gene expression files containing logRatios, FDR, gene annotations, normalized counts, etc. were formatted with R and converted to MS Excel.

Functional analysis of gene lists

Functional enrichment analysis of relevant genes (up- and/or down-regulated genes) was performed with the enricher function of clusterProfiler [28] using the full list of quantified genes as the universal and Gene Ontology (GO) dataset (Biological Process) as the source of functional terms. Genome sequence, gene coordinates and gene annotations were obtained from ENSEMBL [29] and Biomart [30] version GRCh38, release 104. The data discussed in this publication has been deposited in the NCBI Gene Expression Omnibus (GEO) and will be publically accessible through GEO Series accession number GSE240735.

Differential gene expression and gene set enrichment analysis of wild-type and WDM TIA1-stress granules under oxidative stress

Differential expressed gene (DEG) analysis between WT and WDM samples from TIA1-dependent SGs was carried out as described [3]. Significant DEGs were considered at FDR values of <0.01. Gene set enrichment analysis (GSEA) of GO biological processes was performed on significant DEGs (FDR <0.05) using PANTHER (http://pantherdb.org), Enrichr (https://maayanlab.cloud/Enrichr/), and Proteomaps (https://proteomaps.net/) bioinformatics tools. The resulting enriched GO list was filtered at FDR <0.05.

Additional *in silico* analysis of gene lists

The SYnGO-ID conversion tool (https://www.syngoportal.org/convert) tool was used to identify protein/gene IDs into gene identifiers and symbols. Venn diagrams were constructed using the Venny 2.1.0 - Bioinfo GP tool (http://bioinfogp.cnb.csic.es/tools/venny) from the database of SGs, comparing them with

the updated MSGP - Database available online (https://msgp.pt/) and ribonucleoprotein (RNP) granule database available online (https://rnagranuledb.lunenfeld.ca/) and P-bodies database (https://www.uniprot.org/uniprotkb?). The human collection of RBPs was obtained from EuRBPDB -a comprehensive database of RBPs- database available online (http://eurbpdb.gzsys.org.cn/).

Statistical analysis

Data are represented as mean $\pm$ standard error of the means (SEM). Student's t-test (non-paired, different variance and two-tailed) was applied to determine statistical significance between 2 groups. *P*-values of < 0.1 were considered statistically significant. ImageJ/Fiji software was used for immunofluorescence microscopy analysis. To quantify P-bodies and establishing their measurements, we analyzed the particles with a macro setting a threshold (250, 255). We obtained their area, mean, standard deviation, perimeter, Feret and median. Feret's diameter was used to classify P-bodies into three categories: < 1 µm, 1–2 µm or >2 µm. The data are represented as mean $\pm$ SEM. Student's t-test (non-paired, different variance and two-tailed) was applied to determine statistical significance between 2 groups. *P*-values of < 0.5 were considered statistically significant. We also analyzed images to establish Manders' colocalization coefficients, M1 and M2. Colocalization was observed by using two images representing red (P-bodies) and green (GFP-TIA1 SGs) fluorescent channels, allowing for an estimation of the fraction of one molecule colocalizing with the other and *vice versa*. M1 represents fraction of A (GFP-TIA1) overlapping B (P-bodies), and M2 represents fraction of B overlapping A. The values of 0–1 indicate the degree of colocalization. A value of 1 for both channels means a perfect colocalization. Pearson's coefficient was used to assess the correlation of the signals between P-bodies and SGs. A direct relationship between them is observed when r = 1, a lack of relation is r = 0 and if there is an exclusion between samples r = -1.

Legends to Supplementary Figures

Figure S1. Immunopurification of GFP-TIA1aWT/WDM-dependent SGs in FT293 cells under oxidative stress. (A–E) Subcellular fractionation and affinity purification of TIA1-dependent stress granules (SGs) from GFP-TIA1aWT/WDM-expressing FT293 cells under sodium arsenite (NaAsO2) oxidative stress. (A) Schematic diagram of the SG enrichment protocol by differential centrifugation and immunoprecipitation with an anti-GFP antibody coupled to magnetic beads. The method of enrichment of TIA1-dependent SGs was adapted from specific protocols as described previously (see 'Materials and

methods' section). (B and C) Validation of subcellular fractionation of wild-type (WT) (B) and Welander distal myopathy (WDM) (C) GFP-TIA1a-expressing FT293 cells in the absence and the presence of NaAsO2 oxidative stress by western blotting using anti-TIA1 (study target), anti-U2AF2 (nuclear marker), and anti-TUBA (cytoplasmic marker) antibodies. (D and E) Affinity purification of TIA1-dependent SGs from an enriched subcellular fraction. Western blot analysis of immunoprecipitation of ectopic GFP-TIA1 and endogenous TIA1 (eTIA1(a/b)) from an enriched fraction of TIA1-dependent SGs from WT (D) and WDM (E) GFP-TIA1a-expressing FT293 cells. The subcellular fractionation was verified as in B and C panels. Abbreviations: T, total; N, nucleus; SB, cytoplasmic supernatant; GFP-TIA1a, ectopic expression of GFP-TIA1a; eTIA1(a/b), endogenous expression of TIA1 isoforms a and b; U2AF2, U2 small nuclear RNA auxiliary factor 2; and TUBA, alpha tubulin.

Figure S2. Venn diagram displaying numbers and GO enriched categories of DIGs from differential proteomic data generated by label-free quantification in FT293-GFP-TIA1aWT/WDM cells under oxidative stress. (A) Comparison between proteomic data (label-free quantification [LFQ] with significance (S) > 20) and Homo sapiens database of RNA-binding proteins (EuRBPDB) (http://eurbpdb.gzsys.org.cn/). (B and C) Histograms of GO biological processes of shared RBPs (116 genes in B) and non-shared genes (22 genes in C) using the Enrichr tool (https://maayanlab.cloud/Enrichr/). (D) Venn diagram analysis of differential proteomic LFQ (S > 20) data with identified genes in G3BP1-dependent SGs [5]. (E-H). GO biological process analysis of identified genes in immunopurified TIA1 differential proteomic LFQ analysis (S > 20) (138 genes in E), G3BP1-dependent SGs (139 genes in F), shared genes (43 genes in G) and unshared genes (96 genes in H), by Enrichr tool.

Figure S3. Venn diagram displaying numbers and GO enriched categories of DIGs of differential proteomic data generated by label-free quantification from TIA1aWT/WDM-expressing FT293 cells under oxidative stress, and P-bodies database. (A) Venn diagrams of label-free quantification (LFQ) proteomic data, and P-bodies database. (B-E) GO biological process analysis of P-bodies database (255 genes in B), shared genes (37 genes in C), nonshared genes in LFQ proteomic data (101 genes in D) and nonshared genes in P-bodies database (218 genes in E), by Enrichr tool.

Figure S4. Distribution profiling of RNA populations identified in GFP-TIA1aWT/WDM-expressing FT293 cells and GFP-TIA1aWT/WDM-dependent SGs in

proteostasis and under oxidative stress using RNA-Seq analysis. The legend identifies the different classes of RNAs found in massive RNA sequencing approaches.

Figure S5. Transcriptomic data by RNA-seq and Gene Ontology analysis of differentially immunoprecipitated genes in GFP-TIA1a^{WT}-expressing FT293 cells in proteostasis and under oxidative stress. (A-D) Top categories of biological processes associated with differentially immunoprecipitated genes (DIGs). (A) MA plot representations of the distributions of over- (spots in red) and under- (spots in green) expressed genes (-1 > log fold change < 1; FDR < 0.05) in the corresponding combinations indicated in A. (B-D) Histograms of the distributions of over- and under-expressed genes (violet bars in B), only over-expressed genes (red bars in C) and only under-expressed genes (green bars in D) in the corresponding combinations using the GO PANTHER database ($P < 0.05$).

Figure S6. Transcriptomic data by RNA-seq and Gene Ontology analysis of differentially immunoprecipitated genesin GFP-TIA1a^{WDM}-expressing FT293 cells in proteostasis and under oxidative stress. (A-D) Top categories of biological processes associated with differentially immunoprecipitated genes (DIGs). (A) MA plot representations of the distributions of over- (spots in red) and under- (spots in green) expressed genes (-1 > log fold change < 1; FDR < 0.05) in the corresponding combinations indicated in A. (B-D) Histograms of the distributions of over- and under-expressed genes (violet bars in B), only over-expressed genes (red bars in C) and only under-expressed genes (green bars in D) in the corresponding combinations using the GO PANTHER database ($P < 0.05$).

Figure S7. Differential subcellular localization of cytoplasmic markers/proteins in WT and WDM GFP-TIA1a-expressing FT293 cells under oxidative stress by confocal immunofluorescence microscopy. Fluorescence images of GFP-TIA1aWT and GFP-TIA1aWDM-expressing FT293 cells (green) immunolabeled using specific antibodies against ACTB, CNX, CD63, NFE2L2, TOMM20, TUBA and VIM proteins (red). Nuclei were stained with To-Pro3 (blue in merged image). Scale bars (image 60×, zoom 3 and zoom 3 plus crop) represent 10 μm and 2 μm, respectively. Abbreviations: ACTB, beta Actin; CNX, Calnexin; CD63, Lysosomal-associated membrane protein 3; NFE2L2, Nuclear factor erythroid 2-related factor 2; TOMM20, Translocase of outer mitochondrial membrane 20; TUBA, Tubulin alpha; and VIM, Vimentin.

Figure S8. Differential subcellular localization of specific RBPs associated with GFP-TIA1aWT/WDM-dependent SGs under oxidative stress by confocal immunofluorescence microscopy. Fluorescence images showing GFP-TIA1aWT and

GFP-TIA1aWDM-expressing FT293 cells (green) immunolabeled for specific RBPs (red) partially colocalized with TIA1-dependent SGs using specific antibodies against hnRNPA2/B1, hnRNPA1, hnRNPK, PTBP1 and TARDBP. Nuclei were stained with To-Pro3 (blue in merged images). Scale bars (image 60×, zoom 3 and zoom 3 plus crop) represent 10 μm and 2 μm, respectively. Abbreviations: hnRNPA2B1, Heterogeneous nuclear ribonucleoprotein A2/B1; hnRNPA1, Heterogeneous nuclear ribonucleoprotein A1; hnRNPK, Heterogeneous nuclear ribonucleoprotein K; PTBP1, Heterogeneous nuclear ribonucleoprotein polypeptide I; and TARDBP, TAR DNA-binding protein 43.

Figure S9. Differential subcellular localizations of autophagy machinery-associated components in GFP-TIA1aWT/WDM-dependent SGs under oxidative stress by confocal immunofluorescence microscopy. Fluorescence images showing GFP-TIA1aWT and GFP-TIA1aWDM-expressing FT293 cells (green) immunolabeled for specific autophagic components partially localized with TIA1-dependent SGs using specific antibodies against ATG3, ATG7, ATG12, and ATG16L1 proteins. Nuclei were stained with To-Pro3 (blue in merged images). Scale bars (image 60×, zoom 3 and zoom 3 plus crop) represent 10 μm and 2 μm, respectively. Abbreviations: ATG3, Autophagy related 3; ATG7, Autophagy related 7; ATG12, Autophagy related 12; and ATG16L1, Autophagy related 16 like 1.

Figure S10. Enrichment of GFP-TIA1aWT/WDM-dependent SGs and PBs in FT293 cells under oxidative stress. (A–C) Subcellular fractionation of GFP-TIA1aWT/WDM-expressing FT293 cells under sodium arsenite ($NaAsO_2$) oxidative stress. (A) Schematic diagram of the enrichment protocol by differential centrifugation (see Fig. S1). (B and C) Validation of subcellular fractionation of wild-type (WT) (B) and Welander distal myopathy (WDM) (C) GFP-TIA1a-expressing FT293 cells in the absence and the presence of $NaAsO_2$ oxidative stress by western blotting using anti-TIA1, anti-EDC4, anti-DDX6, and anti-U2AF1 antibodies. Abbreviations: T, total; N, nucleus; C, cytoplasmic supernatant; P, 16,800xg pellet; S, post-16,800xg supernatant; GFP-TIA1a, ectopic expression of GFP-TIA1a; EDC4, Enhancer of mRNA decapping; DDX6, DEAD-box helicase 6; and U2AF1, U2 small nuclear RNA auxiliary factor 1.

Figures S11-S14 Original images of western blot analysis.

References

1. Sánchez-Jiménez C, Ludeña MD, Izquierdo JM. T-cell intracellular antigens function as tumor suppressor genes. *Cell Death Dis.* 2015;6(3):e1669. doi.org/10.1038/cddis.2015.43.
2. Carrascoso I, Alcalde J, Sánchez-Jiménez C, et al. T-cell intracellular antigens and Hu antigen R antagonistically modulate mitochondrial activity and dynamics by regulating optic atrophy 1 gene expression. *Mol Cell Biol.* 2017;37(17):e00174–17. doi.org/10.1128/MCB.00174-17.
3. Carrascoso I, Alcalde J, Tabas-Madrid T, et al. Transcriptome-wide analysis links the short-term expression of the b isoforms of TIA proteins to protective proteostasis-mediated cell quiescence response. *PLoS One.* 2018;13(12):e0208526. doi.org/ 10.1371/journal.pone.0208526.
4. Carrascoso I, Sánchez-Jiménez C, Silion E, et al. A heterologous cell model for studying the role of T-cell intracellular antigen 1 in Welander distal myopathy. *Mol Cell Biol.* 2019;39(1):e0029918. doi.org/10.1128/MCB.00299-18.
5. Jain S, Wheeler JR, Walters RW, et al. ATPase-modulated stress granules contain a diverse proteome and substructure. *Cell.* 2016;164(3):487–498. doi.org/10.1016/j.cell.2015.12.038.
6. Souquere S, Mollet S, Kress M, et al. Unravelling the ultrastructure of stress granules and associated P-bodies in human cells. *J Cell Sci.* 2009;122(Pt20):3619–3626. doi.org/ 10.1242/jcs.054437.
7. Wheeler JR, Jain S, Khong A, Parker R. Isolation of yeast and mammalian stress granule cores. *Methods.* 2017;126:12–17. doi.org/10.1016/j.ymeth.2017.04.020.
8. An H, Tan JT, Shelkovnikova TA. Stress granules regulate stress-induced paraspeckle assembly. *J Cell Biol.* 2019;218(12):4127–4140. doi.org/10.1083/jcb.201904098.
9. Moreno ML, Escobar J, Izquierdo-Álvarez A, et al. Disulfide stress: a novel type of oxidative stress in acute inflammation. *Free Rad Biol Med.* 2014;70:265–277. doi.org/10.1016/j.freeradbiomed.2014.01.009.
10. Shevchenko A, Wilm M, Vorm O, Mann M. Mass spectrometric sequencing of proteins silver-stained polyacrylamide gels. *Anal Chem.* 1996;68(5):850–858. doi.org/10.1021/ac950914h.
11. Alonso R, Pisa D, Marina AI, et al. Evidence for fungal infection in cerebrospinal fluid and brain tissue from patients with amyotrophic lateral sclerosis. *Int J Biol Sci.* 2015;11(5):546–558. doi.org/10.7150/ijbs.11084.

12. Jorge I, Casas EM, Villar M, et al. High-sensitivity analysis of specific peptides in complex samples by selected MS/MS ion monitoring and linear ion trap mass spectrometry: application to biological studies. *J Mass Spect.* 2007;42(11):1391–1403. doi.org/ 10.1002/jms.1314.
13. Tran NH, Qiao R, Xin L, et al. Deep learning enables de novo peptide sequencing from data-independent-acquisition mass spectrometry. *Nat Methods.* 2019;16(1):63–66. doi.org/10.1038/s41592-018-0260-3.
14. Tran NH, Zhang X, Xin L, et al. De novo peptide sequencing by deep learning. *Proc Natl Acad Sci.* 2017;114(31):8247–8252. doi.org/10.1073/pnas.1705691114.
15. Tran NH, Rahman MZ, He L, et al. Complete de novo assembly of monoclonal antibody sequences. *Sci Rep.* 2016;6: 31730. doi.org/10.1038/srep31730.
16. Deutsch EW, Bandeira N, Sharma V, et al. The ProteomeXchange consortium in 2020: enabling 'big data' approaches in proteomics. *Nucleic Acids Res.* 2020;48(D1):145–D1152. doi.org/10.1093/nar/gkz984.
17. Perez-Riverol Y, Bai J, Bandla C, et al. The PRIDE database resources in 2022: A Hub for mass spectrometry-based proteomics evidences. *Nucleic Acids Res.* 2022;50(D1): D543–D552. doi.org/10.1093/nar/gkab1038.
18. Clement CC, Wang W, Dzieciatkowska M, et al. Quantitative profiling of the lymph node clearance capacity. *Sci Rep.* 2018;8(1): 11253. doi.org/10.1038/s41598-018-29614-0.
19. Andrews S (Babraham Bioinformatics). https://www.bioinformatics.babraham.ac.uk/projects/fastqc/
20. Bolger AM, Lohse M, Usadel B. Trimmomatic: a flexible trimmer for Illumina sequence data. *Bioinformatics.* 2014;30(15):2114-2120. doi.org/10.1093/bioinformatics/btu170.
21. Dobin A, Davis CA, Schlesinger F, et al. STAR: ultrafast universal RNA-seq aligner. *Bioinformatics* 2013;29(1):15–21. doi.org/10.1093/bioinformatics/bts635.
22. McKenna A, Hanna M, Banks E, et al. The Genome Analysis Toolkit: a MapReduce framework for analyzing next-generation DNA sequencing data. *Genome Res.* 2010;20(9):1297–1303. doi.org/10.1101/gr.107524.110.
23. Li H, Handsaker B, Wysoker A, et al. 1000 Genome Project Data Processing Subgroup. The Sequence Alignment/Map format and SAMtools. *Bioinformatics.* 2009;25(16):2078–2079. doi.org/10.1093/bioinformatics/btp352.

24. Liao Y, Smyth GK, Shi W. The R package Rsubread is easier, faster, cheaper and better for alignment and quantification of RNA sequencing reads. *Nucleic Acids Res.* 2019;47(8):e47. doi.org/10.1093/nar/gkz114.
25. Thorvaldsdóttir H, Robinson JT, Mesirov JP. Integrative Genomics Viewer (IGV): high- performance genomics data visualization and exploration. *Brief Bioinform.* 2013;14(2):178–192. doi.org/10.1093/bib/bbs017.
26. Love MI, Huber W, Anders S. Moderated estimation of fold change and dispersion for RNA-seq data with DESeq2. *Genome Biol.* 2014;15(12):550. doi.org/10.1186/s13059-014-0550-8.
27. Zhu A, Ibrahim JG, Love MI. Heavy-tailed prior distributions for sequence count data: removing the noise and preserving large differences. *Bioinformatics.* 2019;35(12): 2084–2092. doi.org/10.1093/bioinformatics/bty895.
28. Yu G, Wang L, Han Y, He Q. clusterProfiler: an R package for comparing biological themes among gene clusters. *OMICS.* 2012;16(5):284–287. doi.org/10.1089/omi.2011.0118.
29. Yates AD, Achuthan P, Akanni W, et al. Ensembl 2020. *Nucleic Acids Res.* 2020;48(D1): D682–D688. Doi.org/10.1093/nar/gkz966.
30. Kinsella RJ, Kähäri A, Haider S, et al. Ensembl BioMarts: a hub for data retrieval across taxonomic space. *Database (Oxford).* 2011;2011:bar030. doi.org/10.1093/database/bar030.

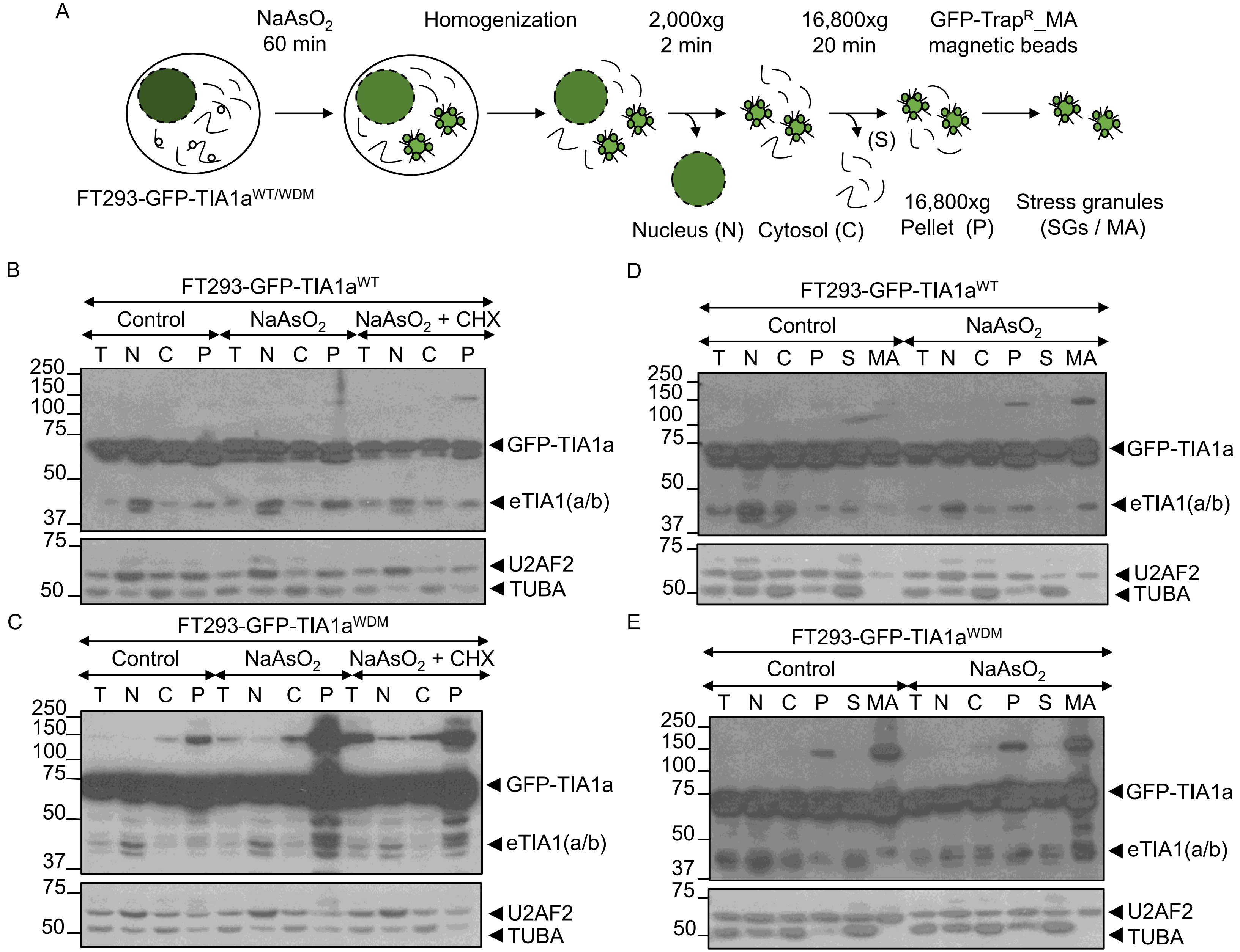
A
NaAsO2 60 min
Homogenization
2,000xg 2 min
16,800xg 20 min
GFP-TrapR_MA magnetic beads
FT293-GFP-TIA1aWT/WDM
(S)
Nucleus (N)
Cytosol (C)
16,800xg Pellet (P)
Stress granules (SGs / MA)
B
FT293-GFP-TIA1aWT
Control
NaAsO2
NaAsO2 + CHX
T N C P T N C P T N C P
250
150
100
75
50
37
GFP-TIA1a
eTIA1(a/b)
U2AF2
TUBA
C
FT293-GFP-TIA1aWDM
Control
NaAsO2
NaAsO2 + CHX
T N C P T N C P T N C P
D
FT293-GFP-TIA1aWT
Control
NaAsO2
T N C P S MA T N C P S MA
E
FT293-GFP-TIA1aWDM
Control
NaAsO2
T N C P S MA T N C P S MA

Figure S1

Figure S2

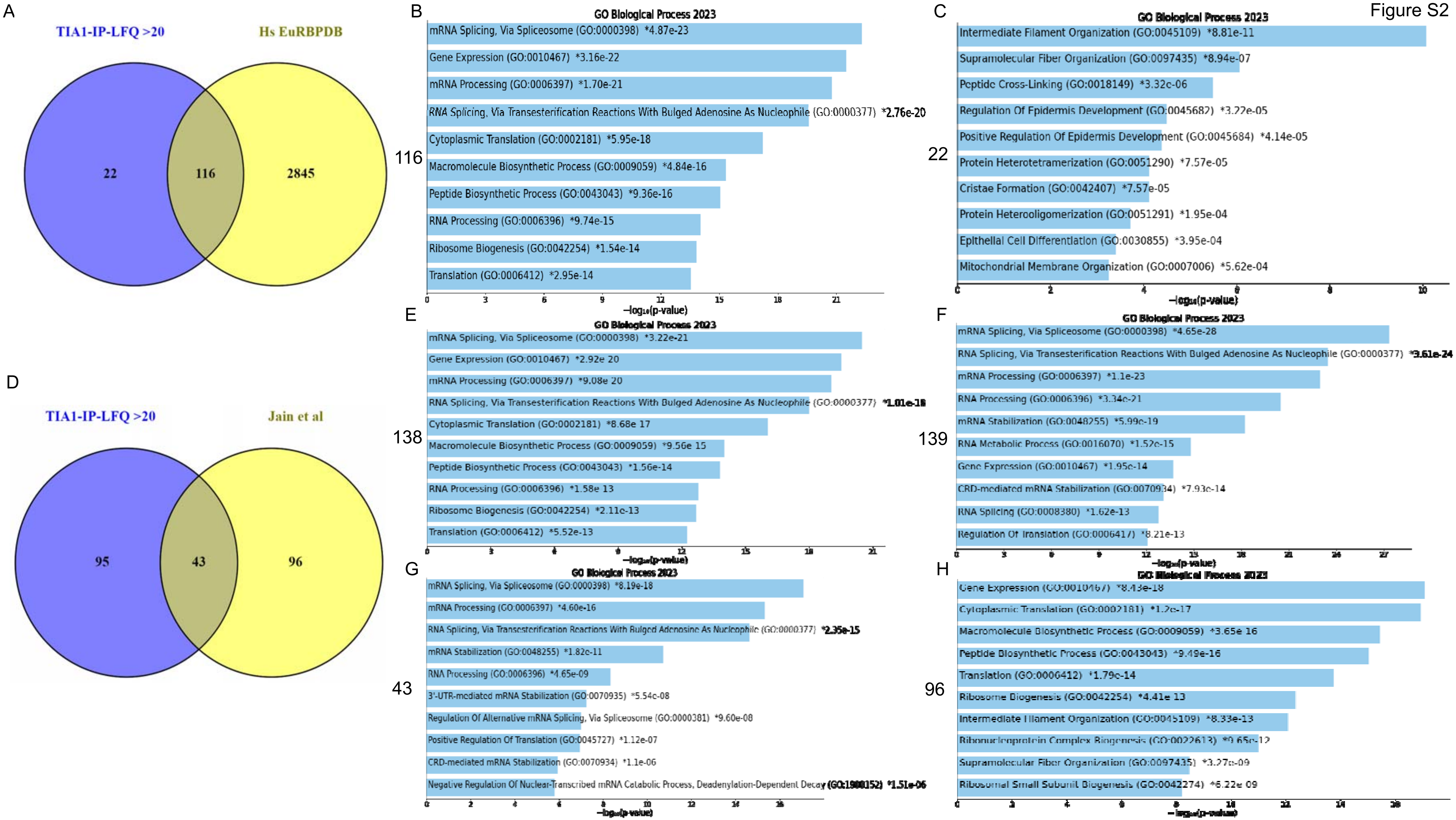

A
TIA1-IP-LFQ >20
Hs EuRBPDB
22
116
2845
B
GO Biological Process 2023
mRNA Splicing, Via Spliceosome (GO:0000398) *4.87e-23
Gene Expression (GO:0010467) *3.16e-22
mRNA Processing (GO:0006397) *1.70e-21
RNA Splicing, Via Transesterification Reactions With Bulged Adenosine As Nucleophile (GO:0000377) *2.76e-20
Cytoplasmic Translation (GO:0002181) *5.95e-18
Macromolecule Biosynthetic Process (GO:0009059) *4.84e-16
Peptide Biosynthetic Process (GO:0043043) *9.36e-16
RNA Processing (GO:0006396) *9.74e-15
Ribosome Biogenesis (GO:0042254) *1.54e-14
Translation (GO:0006412) *2.95e-14
116
−log10(p-value)
C
GO Biological Process 2023
Intermediate Filament Organization (GO:0045109) *8.81e-11
Supramolecular Fiber Organization (GO:0097435) *8.94e-07
Peptide Cross-Linking (GO:0018149) *3.32e-06
Regulation Of Epidermis Development (GO:0045682) *3.22e-05
Positive Regulation Of Epidermis Development (GO:0045684) *4.14e-05
Protein Heterotetramerization (GO:0051290) *7.57e-05
Cristae Formation (GO:0042407) *7.57e-05
Protein Heterooligomerization (GO:0051291) *1.95e-04
Epithelial Cell Differentiation (GO:0030855) *3.95e-04
Mitochondrial Membrane Organization (GO:0007006) *5.62e-04
22
−log10(p-value)
D
TIA1-IP-LFQ >20
Jain et al
95
43
96
E
GO Biological Process 2023
mRNA Splicing, Via Spliceosome (GO:0000398) *3.22e-21
Gene Expression (GO:0010467) *2.92e 20
mRNA Processing (GO:0006397) *9.08e 20
RNA Splicing, Via Transesterification Reactions With Bulged Adenosine As Nucleophile (GO:0000377) *1.01e-18
Cytoplasmic Translation (GO:0002181) *8.68e 17
Macromolecule Biosynthetic Process (GO:0009059) *9.56e 15
Peptide Biosynthetic Process (GO:0043043) *1.56e-14
RNA Processing (GO:0006396) *1.58e 13
Ribosome Biogenesis (GO:0042254) *2.11e-13
Translation (GO:0006412) *5.52e-13
138
−log10(p-value)
F
GO Biological Process 2023
mRNA Splicing, Via Spliceosome (GO:0000398) *4.65e-28
RNA Splicing, Via Transesterification Reactions With Bulged Adenosine As Nucleophile (GO:0000377) *3.61e-24
mRNA Processing (GO:0006397) *1.1e-23
RNA Processing (GO:0006396) *3.34e-21
mRNA Stabilization (GO:0048255) *5.99e-19
RNA Metabolic Process (GO:0016070) *1.52e-15
Gene Expression (GO:0010467) *1.95e-14
CRD-mediated mRNA Stabilization (GO:0070934) *7.93e-14
RNA Splicing (GO:0008380) *1.62e-13
Regulation Of Translation (GO:0006417) *8.21e-13
139
−log10(p-value)
G
GO Biological Process 2023
mRNA Splicing, Via Spliceosome (GO:0000398) *8.19e-18
mRNA Processing (GO:0006397) *4.60e-16
RNA Splicing, Via Transesterification Reactions With Bulged Adenosine As Nucleophile (GO:0000377) *2.35e-15
mRNA Stabilization (GO:0048255) *1.82e-11
RNA Processing (GO:0006396) *4.65e-09
3'-UTR-mediated mRNA Stabilization (GO:0070935) *5.54e-08
Regulation Of Alternative mRNA Splicing, Via Spliceosome (GO:0000381) *9.60e-08
Positive Regulation Of Translation (GO:0045727) *1.12e-07
CRD-mediated mRNA Stabilization (GO:0070934) *1.1e-06
Negative Regulation Of Nuclear-Transcribed mRNA Catabolic Process, Deadenylation-Dependent Decay (GO:1900152) *1.51e-06
43
−log10(p-value)
H
GO Biological Process 2023
Gene Expression (GO:0010467) *8.43e-18
Cytoplasmic Translation (GO:0002181) *1.2e-17
Macromolecule Biosynthetic Process (GO:0009059) *3.65e 16
Peptide Biosynthetic Process (GO:0043043) *9.49e-16
Translation (GO:0006412) *1.79e-14
Ribosome Biogenesis (GO:0042254) *4.41e 13
Intermediate Filament Organization (GO:0045109) *8.33e-13
Ribonucleoprotein Complex Biogenesis (GO:0022613) *9.65e-12
Supramolecular Fiber Organization (GO:0097435) *3.27e-09
Ribosomal Small Subunit Biogenesis (GO:0042274) *6.22e 09
96
−log10(p-value)

Figure S3

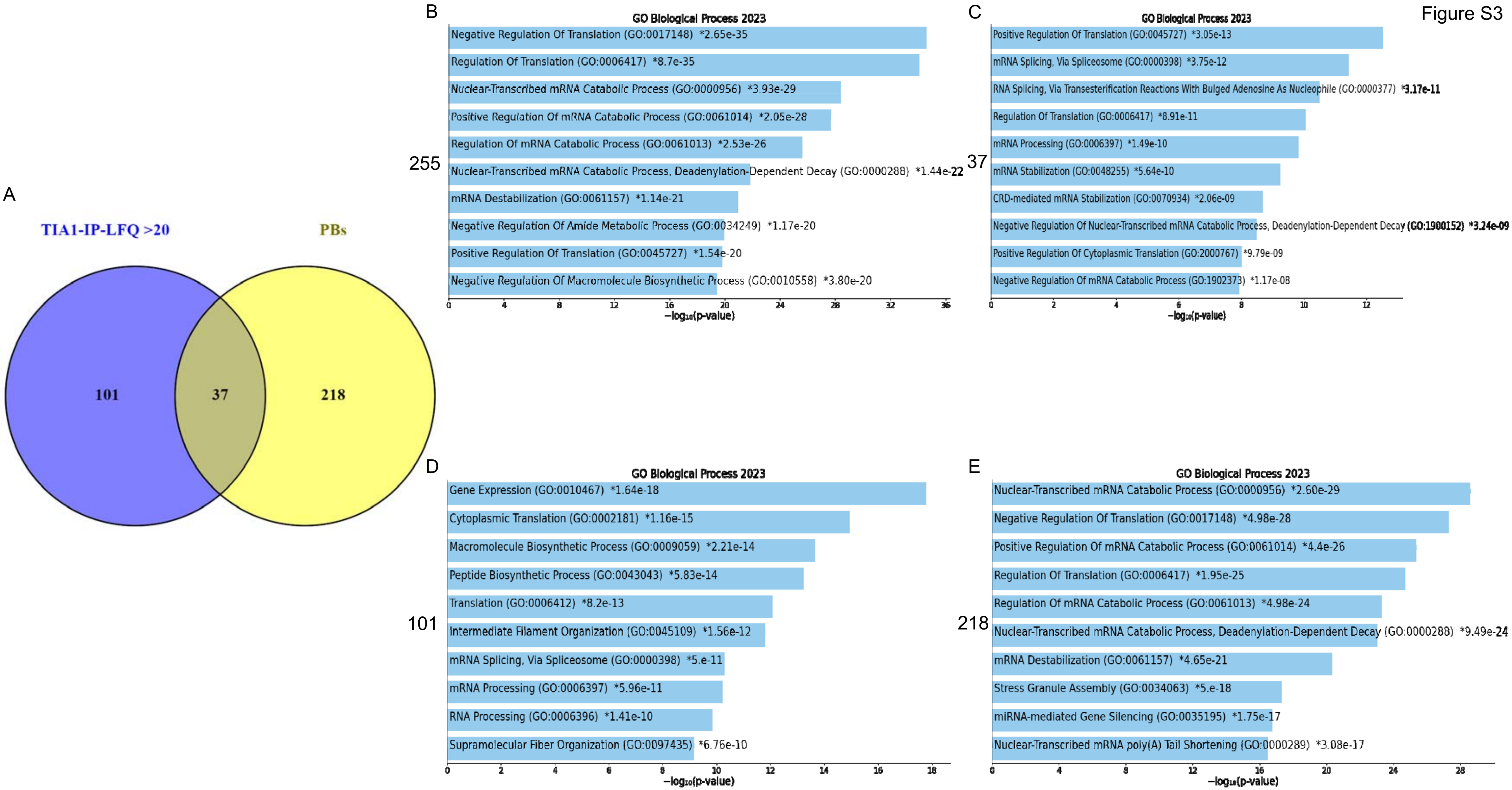
A
TIA1-IP-LFQ >20
PBs
101
37
218
B
GO Biological Process 2023
255
Negative Regulation Of Translation (GO:0017148) *2.65e-35
Regulation Of Translation (GO:0006417) *8.7e-35
Nuclear-Transcribed mRNA Catabolic Process (GO:0000956) *3.93e-29
Positive Regulation Of mRNA Catabolic Process (GO:0061014) *2.05e-28
Regulation Of mRNA Catabolic Process (GO:0061013) *2.53e-26
Nuclear-Transcribed mRNA Catabolic Process, Deadenylation-Dependent Decay (GO:0000288) *1.44e-22
mRNA Destabilization (GO:0061157) *1.14e-21
Negative Regulation Of Amide Metabolic Process (GO:0034249) *1.17e-20
Positive Regulation Of Translation (GO:0045727) *1.54e-20
Negative Regulation Of Macromolecule Biosynthetic Process (GO:0010558) *3.80e-20
0 4 8 12 16 20 24 28 32 36
−log₁₀(p-value)
C
GO Biological Process 2023
37
Positive Regulation Of Translation (GO:0045727) *3.05e-13
mRNA Splicing, Via Spliceosome (GO:0000398) *3.75e-12
RNA Splicing, Via Transesterification Reactions With Bulged Adenosine As Nucleophile (GO:0000377) *3.17e-11
Regulation Of Translation (GO:0006417) *8.91e-11
mRNA Processing (GO:0006397) *1.49e-10
mRNA Stabilization (GO:0048255) *5.64e-10
CRD-mediated mRNA Stabilization (GO:0070934) *2.06e-09
Negative Regulation Of Nuclear-Transcribed mRNA Catabolic Process, Deadenylation-Dependent Decay (GO:1900152) *3.24e-09
Positive Regulation Of Cytoplasmic Translation (GO:2000767) *9.79e-09
Negative Regulation Of mRNA Catabolic Process (GO:1902373) *1.17e-08
0 2 4 6 8 10 12
−log₁₀(p-value)
D
GO Biological Process 2023
101
Gene Expression (GO:0010467) *1.64e-18
Cytoplasmic Translation (GO:0002181) *1.16e-15
Macromolecule Biosynthetic Process (GO:0009059) *2.21e-14
Peptide Biosynthetic Process (GO:0043043) *5.83e-14
Translation (GO:0006412) *8.2e-13
Intermediate Filament Organization (GO:0045109) *1.56e-12
mRNA Splicing, Via Spliceosome (GO:0000398) *5.e-11
mRNA Processing (GO:0006397) *5.96e-11
RNA Processing (GO:0006396) *1.41e-10
Supramolecular Fiber Organization (GO:0097435) *6.76e-10
0 2 4 6 8 10 12 14 16 18
−log₁₀(p-value)
E
GO Biological Process 2023
218
Nuclear-Transcribed mRNA Catabolic Process (GO:0000956) *2.60e-29
Negative Regulation Of Translation (GO:0017148) *4.98e-28
Positive Regulation Of mRNA Catabolic Process (GO:0061014) *4.4e-26
Regulation Of Translation (GO:0006417) *1.95e-25
Regulation Of mRNA Catabolic Process (GO:0061013) *4.98e-24
Nuclear-Transcribed mRNA Catabolic Process, Deadenylation-Dependent Decay (GO:0000288) *9.49e-24
mRNA Destabilization (GO:0061157) *4.65e-21
Stress Granule Assembly (GO:0034063) *5.e-18
miRNA-mediated Gene Silencing (GO:0035195) *1.75e-17
Nuclear-Transcribed mRNA poly(A) Tail Shortening (GO:0000289) *3.08e-17
0 4 8 12 16 20 24 28
−log₁₀(p-value)

Figure S4

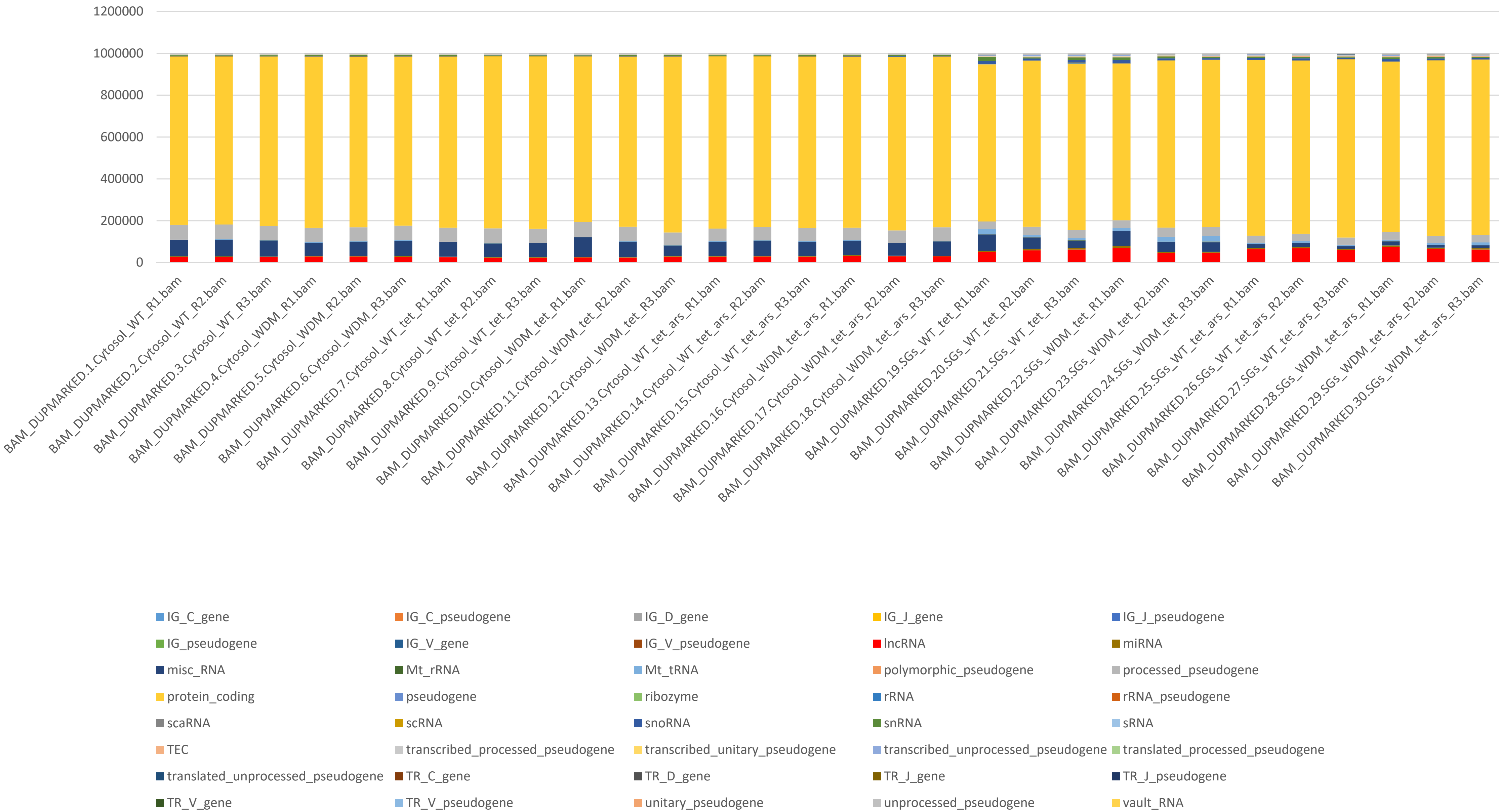

1200000
1000000
800000
600000
400000
200000
0
BAM_DUPMARKED.1.Cytosol_WT_R1.bam
BAM_DUPMARKED.2.Cytosol_WT_R2.bam
BAM_DUPMARKED.3.Cytosol_WT_R3.bam
BAM_DUPMARKED.4.Cytosol_WDM_R1.bam
BAM_DUPMARKED.5.Cytosol_WDM_R2.bam
BAM_DUPMARKED.6.Cytosol_WDM_R3.bam
BAM_DUPMARKED.7.Cytosol_WT_tet_R1.bam
BAM_DUPMARKED.8.Cytosol_WT_tet_R2.bam
BAM_DUPMARKED.9.Cytosol_WT_tet_R3.bam
BAM_DUPMARKED.10.Cytosol_WDM_tet_R1.bam
BAM_DUPMARKED.11.Cytosol_WDM_tet_R2.bam
BAM_DUPMARKED.12.Cytosol_WDM_tet_R3.bam
BAM_DUPMARKED.13.Cytosol_WT_tet_ars_R1.bam
BAM_DUPMARKED.14.Cytosol_WT_tet_ars_R2.bam
BAM_DUPMARKED.15.Cytosol_WT_tet_ars_R3.bam
BAM_DUPMARKED.16.Cytosol_WDM_tet_ars_R1.bam
BAM_DUPMARKED.17.Cytosol_WDM_tet_ars_R2.bam
BAM_DUPMARKED.18.Cytosol_WDM_tet_ars_R3.bam
BAM_DUPMARKED.19.SGs_WT_tet_R1.bam
BAM_DUPMARKED.20.SGs_WT_tet_R2.bam
BAM_DUPMARKED.21.SGs_WT_tet_R3.bam
BAM_DUPMARKED.22.SGs_WDM_tet_R1.bam
BAM_DUPMARKED.23.SGs_WDM_tet_R2.bam
BAM_DUPMARKED.24.SGs_WDM_tet_R3.bam
BAM_DUPMARKED.25.SGs_WT_tet_ars_R1.bam
BAM_DUPMARKED.26.SGs_WT_tet_ars_R2.bam
BAM_DUPMARKED.27.SGs_WT_tet_ars_R3.bam
BAM_DUPMARKED.28.SGs_WDM_tet_ars_R1.bam
BAM_DUPMARKED.29.SGs_WDM_tet_ars_R2.bam
BAM_DUPMARKED.30.SGs_WDM_tet_ars_R3.bam
IG_C_gene
IG_C_pseudogene
IG_D_gene
IG_J_gene
IG_J_pseudogene
IG_pseudogene
IG_V_gene
IG_V_pseudogene
lncRNA
miRNA
misc_RNA
Mt_rRNA
Mt_tRNA
polymorphic_pseudogene
processed_pseudogene
protein_coding
pseudogene
ribozyme
rRNA
rRNA_pseudogene
scaRNA
scRNA
snoRNA
snRNA
sRNA
TEC
transcribed_processed_pseudogene
transcribed_unitary_pseudogene
transcribed_unprocessed_pseudogene
translated_processed_pseudogene
translated_unprocessed_pseudogene
TR_C_gene
TR_D_gene
TR_J_gene
TR_J_pseudogene
TR_V_gene
TR_V_pseudogene
unitary_pseudogene
unprocessed_pseudogene
vault_RNA

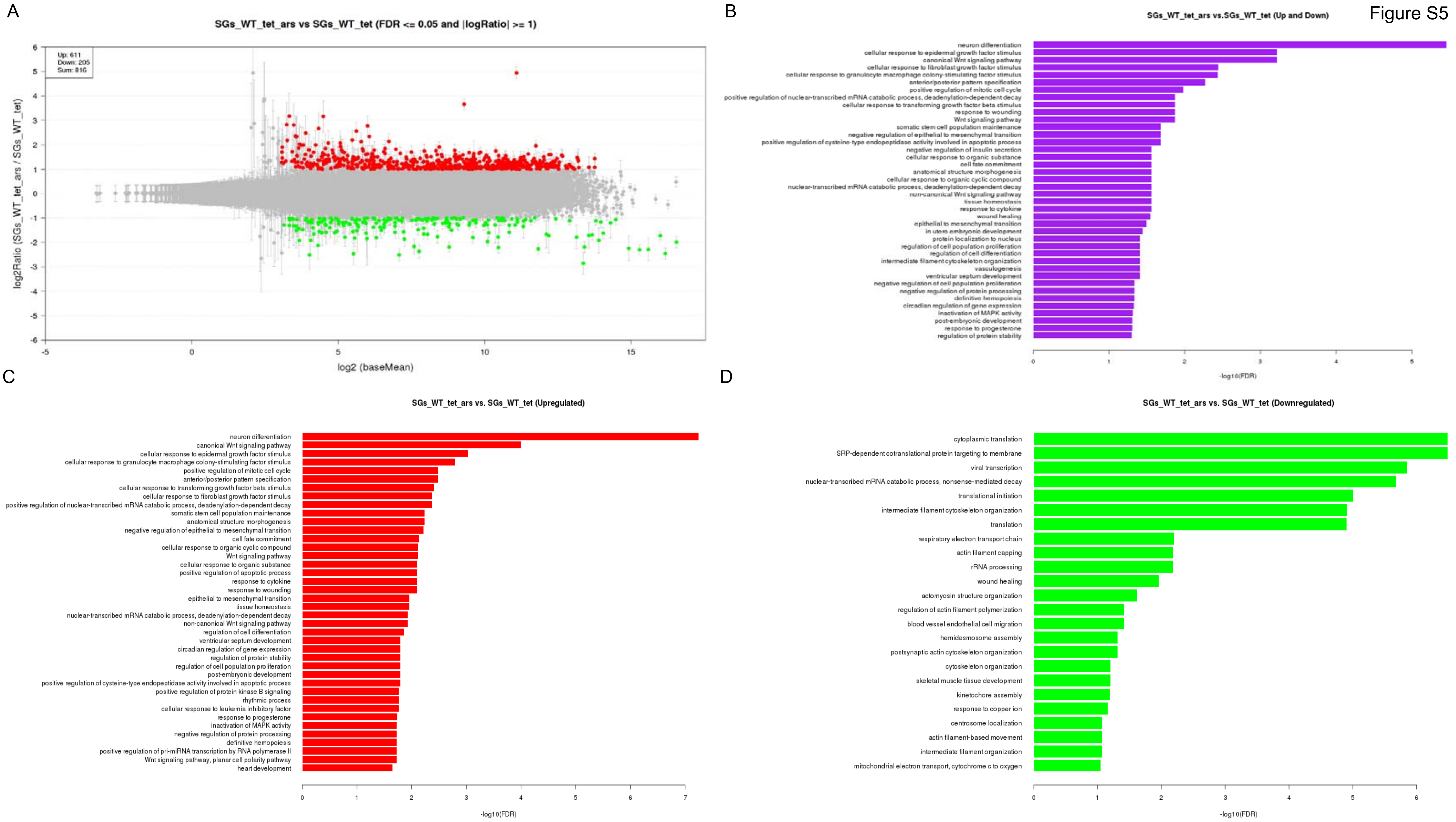
A
SGs_WT_tet_ars vs SGs_WT_tet (FDR <= 0.05 and |logRatio| >= 1)
Up: 611
Down: 205
Sum: 816
log2Ratio (SGs_WT_tet_ars / SGs_WT_tet)
log2 (baseMean)
B
SGs_WT_tet_ars vs.SGs_WT_tet (Up and Down)
neuron differentiation
cellular response to epidermal growth factor stimulus
canonical Wnt signaling pathway
cellular response to fibroblast growth factor stimulus
cellular response to granulocyte macrophage colony-stimulating factor stimulus
anterior/posterior pattern specification
positive regulation of mitotic cell cycle
positive regulation of nuclear-transcribed mRNA catabolic process, deadenylation-dependent decay
cellular response to transforming growth factor beta stimulus
response to wounding
Wnt signaling pathway
somatic stem cell population maintenance
negative regulation of epithelial to mesenchymal transition
positive regulation of cysteine-type endopeptidase activity involved in apoptotic process
negative regulation of insulin secretion
cellular response to organic substance
cell fate commitment
anatomical structure morphogenesis
cellular response to organic cyclic compound
nuclear-transcribed mRNA catabolic process, deadenylation-dependent decay
non-canonical Wnt signaling pathway
tissue homeostasis
response to cytokine
wound healing
epithelial to mesenchymal transition
in utero embryonic development
protein localization to nucleus
regulation of cell population proliferation
regulation of cell differentiation
intermediate filament cytoskeleton organization
vasculogenesis
ventricular septum development
negative regulation of cell population proliferation
negative regulation of protein processing
definitive hemopoiesis
circadian regulation of gene expression
inactivation of MAPK activity
post-embryonic development
response to progesterone
regulation of protein stability
-log10(FDR)
C
SGs_WT_tet_ars vs. SGs_WT_tet (Upregulated)
neuron differentiation
canonical Wnt signaling pathway
cellular response to epidermal growth factor stimulus
cellular response to granulocyte macrophage colony-stimulating factor stimulus
positive regulation of mitotic cell cycle
anterior/posterior pattern specification
cellular response to transforming growth factor beta stimulus
cellular response to fibroblast growth factor stimulus
positive regulation of nuclear-transcribed mRNA catabolic process, deadenylation-dependent decay
somatic stem cell population maintenance
anatomical structure morphogenesis
negative regulation of epithelial to mesenchymal transition
cell fate commitment
cellular response to organic cyclic compound
Wnt signaling pathway
cellular response to organic substance
positive regulation of apoptotic process
response to cytokine
response to wounding
epithelial to mesenchymal transition
tissue homeostasis
nuclear-transcribed mRNA catabolic process, deadenylation-dependent decay
non-canonical Wnt signaling pathway
regulation of cell differentiation
ventricular septum development
circadian regulation of gene expression
regulation of protein stability
regulation of cell population proliferation
post-embryonic development
positive regulation of cysteine-type endopeptidase activity involved in apoptotic process
positive regulation of protein kinase B signaling
rhythmic process
cellular response to leukemia inhibitory factor
response to progesterone
inactivation of MAPK activity
negative regulation of protein processing
definitive hemopoiesis
positive regulation of pri-miRNA transcription by RNA polymerase II
Wnt signaling pathway, planar cell polarity pathway
heart development
-log10(FDR)
D
SGs_WT_tet_ars vs. SGs_WT_tet (Downregulated)
cytoplasmic translation
SRP-dependent cotranslational protein targeting to membrane
viral transcription
nuclear-transcribed mRNA catabolic process, nonsense-mediated decay
translational initiation
intermediate filament cytoskeleton organization
translation
respiratory electron transport chain
actin filament capping
rRNA processing
wound healing
actomyosin structure organization
regulation of actin filament polymerization
blood vessel endothelial cell migration
hemidesmosome assembly
postsynaptic actin cytoskeleton organization
cytoskeleton organization
skeletal muscle tissue development
kinetochore assembly
response to copper ion
centrosome localization
actin filament-based movement
intermediate filament organization
mitochondrial electron transport, cytochrome c to oxygen
-log10(FDR)

Figure S5

Figure S6

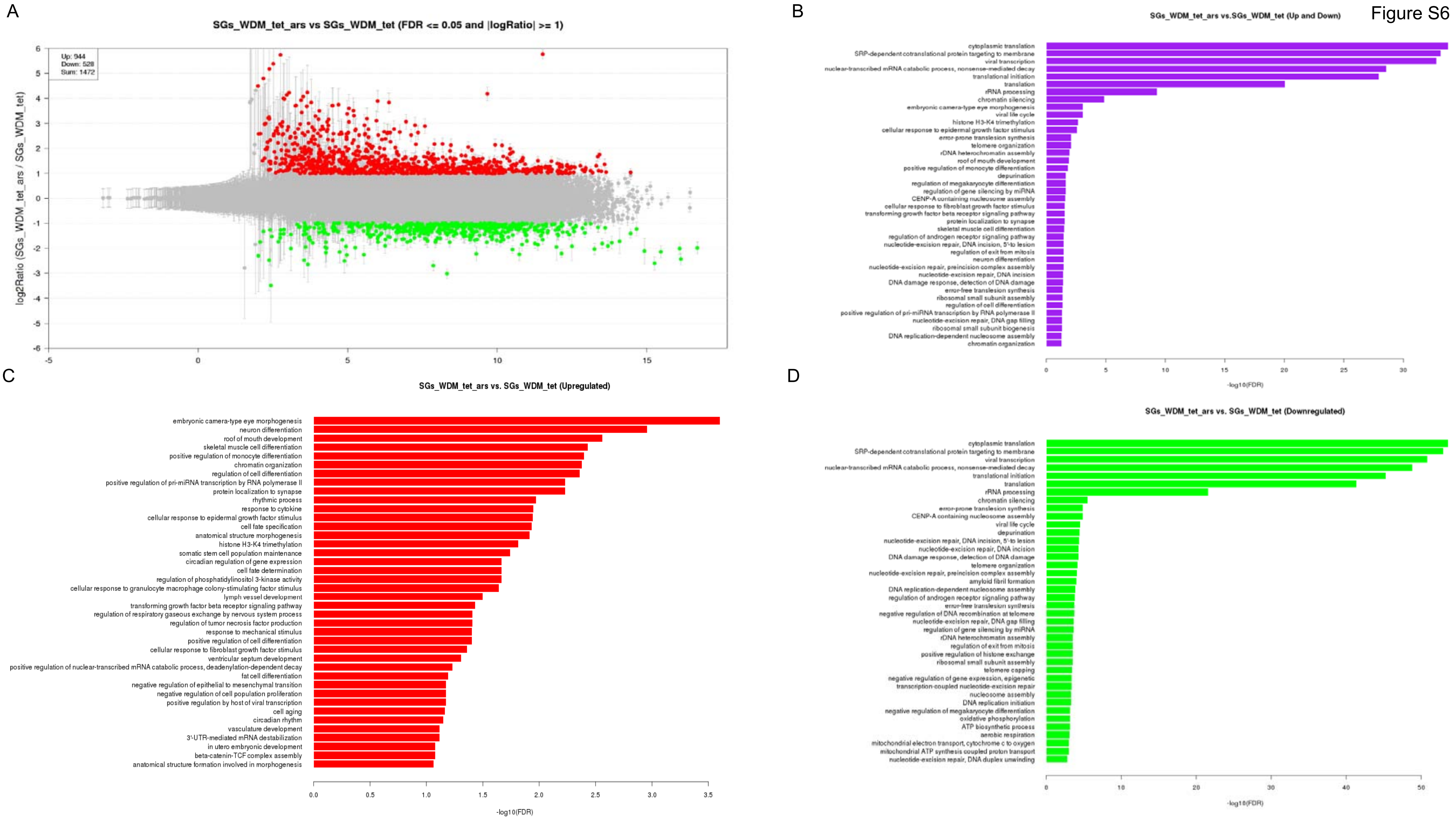
A
SGs_WDM_tet_ars vs SGs_WDM_tet (FDR <= 0.05 and |logRatio| >= 1)
Up: 944
Down: 528
Sum: 1472
log2Ratio (SGs_WDM_tet_ars / SGs_WDM_tet)
6
5
4
3
2
1
0
-1
-2
-3
-4
-5
-6
-5
0
5
10
15
B
SGs_WDM_tet_ars vs.SGs_WDM_tet (Up and Down)
cytoplasmic translation
SRP-dependent cotranslational protein targeting to membrane
viral transcription
nuclear-transcribed mRNA catabolic process, nonsense-mediated decay
translational initiation
translation
rRNA processing
chromatin silencing
embryonic camera-type eye morphogenesis
viral life cycle
histone H3-K4 trimethylation
cellular response to epidermal growth factor stimulus
error-prone translesion synthesis
telomere organization
rDNA heterochromatin assembly
roof of mouth development
positive regulation of monocyte differentiation
depurination
regulation of megakaryocyte differentiation
regulation of gene silencing by miRNA
CENP-A containing nucleosome assembly
cellular response to fibroblast growth factor stimulus
transforming growth factor beta receptor signaling pathway
protein localization to synapse
skeletal muscle cell differentiation
regulation of androgen receptor signaling pathway
nucleotide-excision repair, DNA incision, 5'-to lesion
regulation of exit from mitosis
neuron differentiation
nucleotide-excision repair, preincision complex assembly
nucleotide-excision repair, DNA incision
DNA damage response, detection of DNA damage
error-free translesion synthesis
ribosomal small subunit assembly
regulation of cell differentiation
positive regulation of pri-miRNA transcription by RNA polymerase II
nucleotide-excision repair, DNA gap filling
ribosomal small subunit biogenesis
DNA replication-dependent nucleosome assembly
chromatin organization
0
5
10
15
20
25
30
-log10(FDR)
C
SGs_WDM_tet_ars vs. SGs_WDM_tet (Upregulated)
embryonic camera-type eye morphogenesis
neuron differentiation
roof of mouth development
skeletal muscle cell differentiation
positive regulation of monocyte differentiation
chromatin organization
regulation of cell differentiation
positive regulation of pri-miRNA transcription by RNA polymerase II
protein localization to synapse
rhythmic process
response to cytokine
cellular response to epidermal growth factor stimulus
cell fate specification
anatomical structure morphogenesis
histone H3-K4 trimethylation
somatic stem cell population maintenance
circadian regulation of gene expression
cell fate determination
regulation of phosphatidylinositol 3-kinase activity
cellular response to granulocyte macrophage colony-stimulating factor stimulus
lymph vessel development
transforming growth factor beta receptor signaling pathway
regulation of respiratory gaseous exchange by nervous system process
regulation of tumor necrosis factor production
response to mechanical stimulus
positive regulation of cell differentiation
cellular response to fibroblast growth factor stimulus
ventricular septum development
positive regulation of nuclear-transcribed mRNA catabolic process, deadenylation-dependent decay
fat cell differentiation
negative regulation of epithelial to mesenchymal transition
negative regulation of cell population proliferation
positive regulation by host of viral transcription
cell aging
circadian rhythm
vasculature development
3'-UTR-mediated mRNA destabilization
in utero embryonic development
beta-catenin-TCF complex assembly
anatomical structure formation involved in morphogenesis
0.0
0.5
1.0
1.5
2.0
2.5
3.0
3.5
-log10(FDR)
D
SGs_WDM_tet_ars vs. SGs_WDM_tet (Downregulated)
cytoplasmic translation
SRP-dependent cotranslational protein targeting to membrane
viral transcription
nuclear-transcribed mRNA catabolic process, nonsense-mediated decay
translational initiation
translation
rRNA processing
chromatin silencing
error-prone translesion synthesis
CENP-A containing nucleosome assembly
viral life cycle
depurination
nucleotide-excision repair, DNA incision, 5'-to lesion
nucleotide-excision repair, DNA incision
DNA damage response, detection of DNA damage
telomere organization
nucleotide-excision repair, preincision complex assembly
amyloid fibril formation
DNA replication-dependent nucleosome assembly
regulation of androgen receptor signaling pathway
error-free translesion synthesis
negative regulation of DNA recombination at telomere
nucleotide-excision repair, DNA gap filling
regulation of gene silencing by miRNA
rDNA heterochromatin assembly
regulation of exit from mitosis
positive regulation of histone exchange
ribosomal small subunit assembly
telomere capping
negative regulation of gene expression, epigenetic
transcription-coupled nucleotide-excision repair
nucleosome assembly
DNA replication initiation
negative regulation of megakaryocyte differentiation
oxidative phosphorylation
ATP biosynthetic process
aerobic respiration
mitochondrial electron transport, cytochrome c to oxygen
mitochondrial ATP synthesis coupled proton transport
nucleotide-excision repair, DNA duplex unwinding
0
10
20
30
40
50
-log10(FDR)

Figure S7

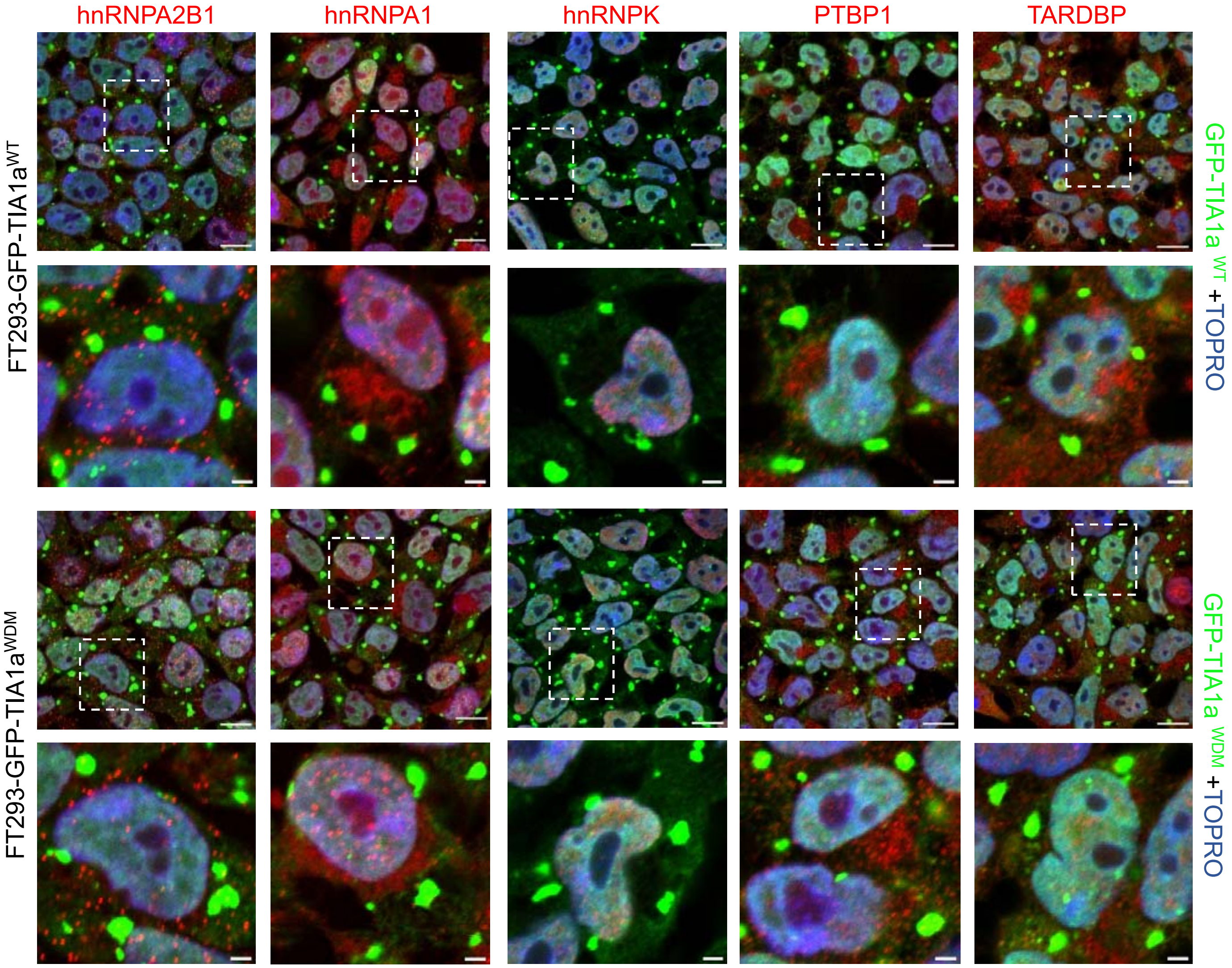

hnRNPA2B1
hnRNPA1
hnRNPK
PTBP1
TARDBP
FT293-GFP-TIA1a WT
GFP-TIA1a WT +TOPRO
FT293-GFP-TIA1a WDM
GFP-TIA1a WDM +TOPRO

Figure S8

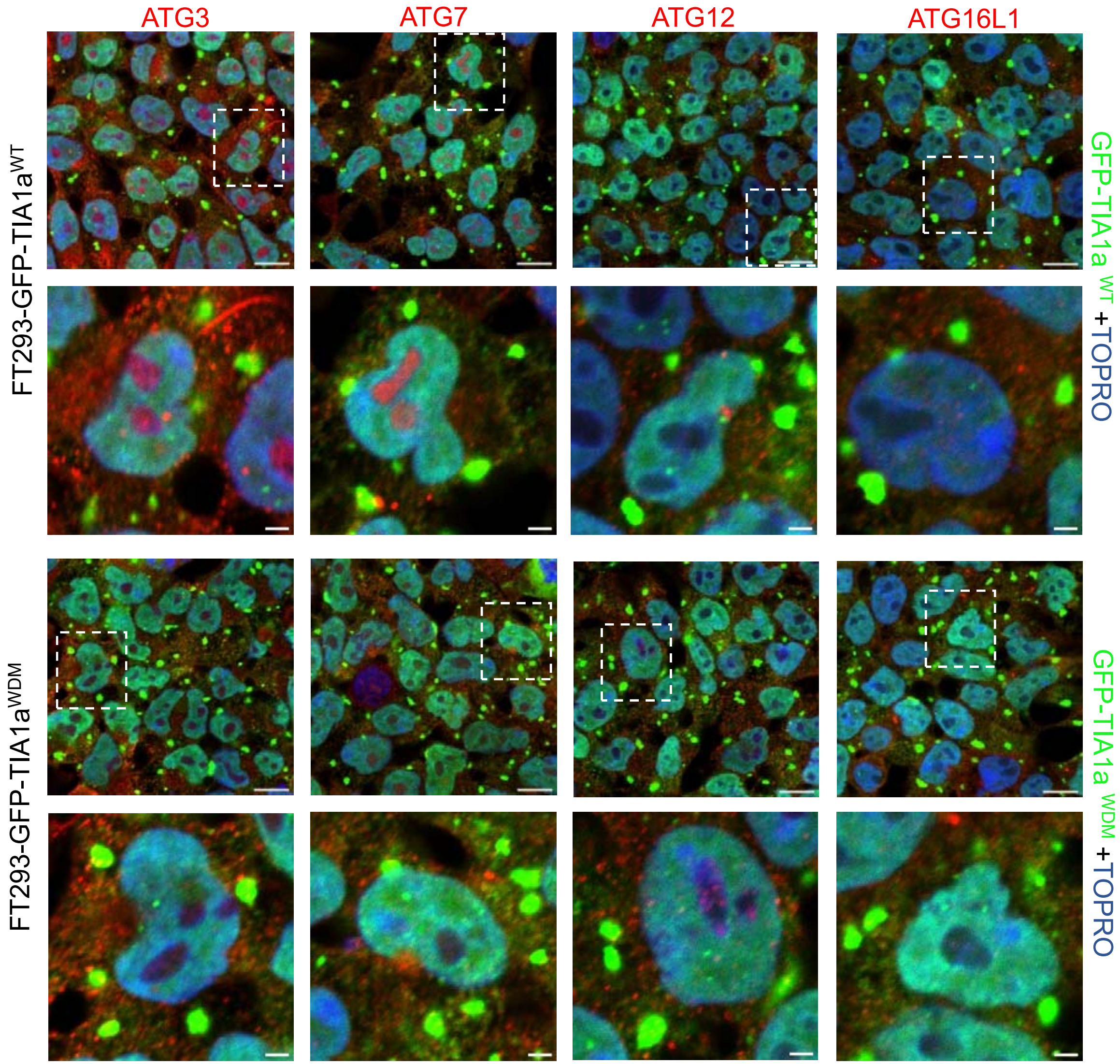

ATG3
ATG7
ATG12
ATG16L1
FT293-GFP-TIA1aWT
FT293-GFP-TIA1aWDM
GFP-TIA1a WT +TOPRO
GFP-TIA1a WDM +TOPRO

Figure S9

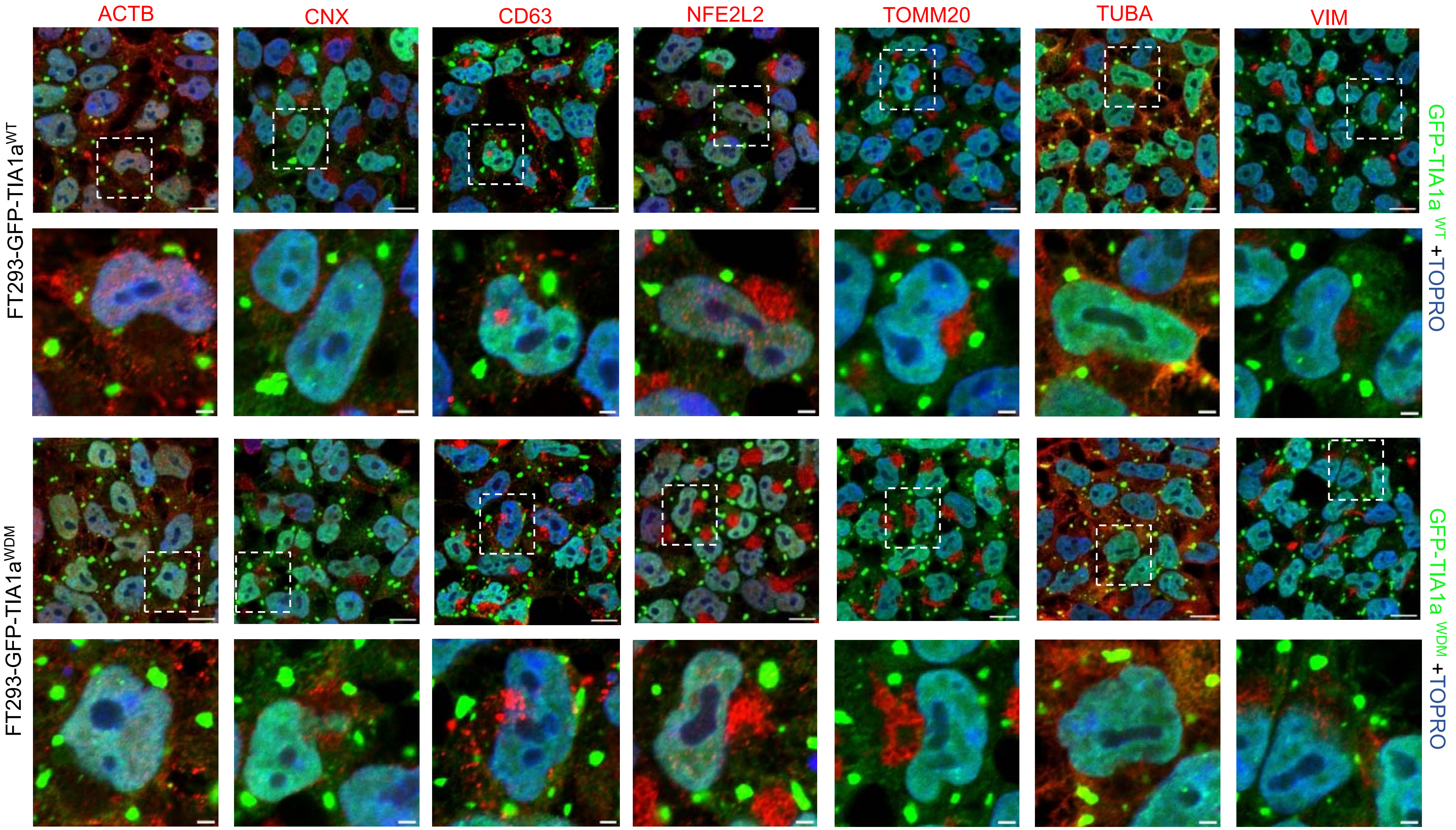

ACTB
CNX
CD63
NFE2L2
TOMM20
TUBA
VIM
FT293-GFP-TIA1aWT
FT293-GFP-TIA1aWDM
GFP-TIA1a WT +TOPRO
GFP-TIA1a WDM +TOPRO

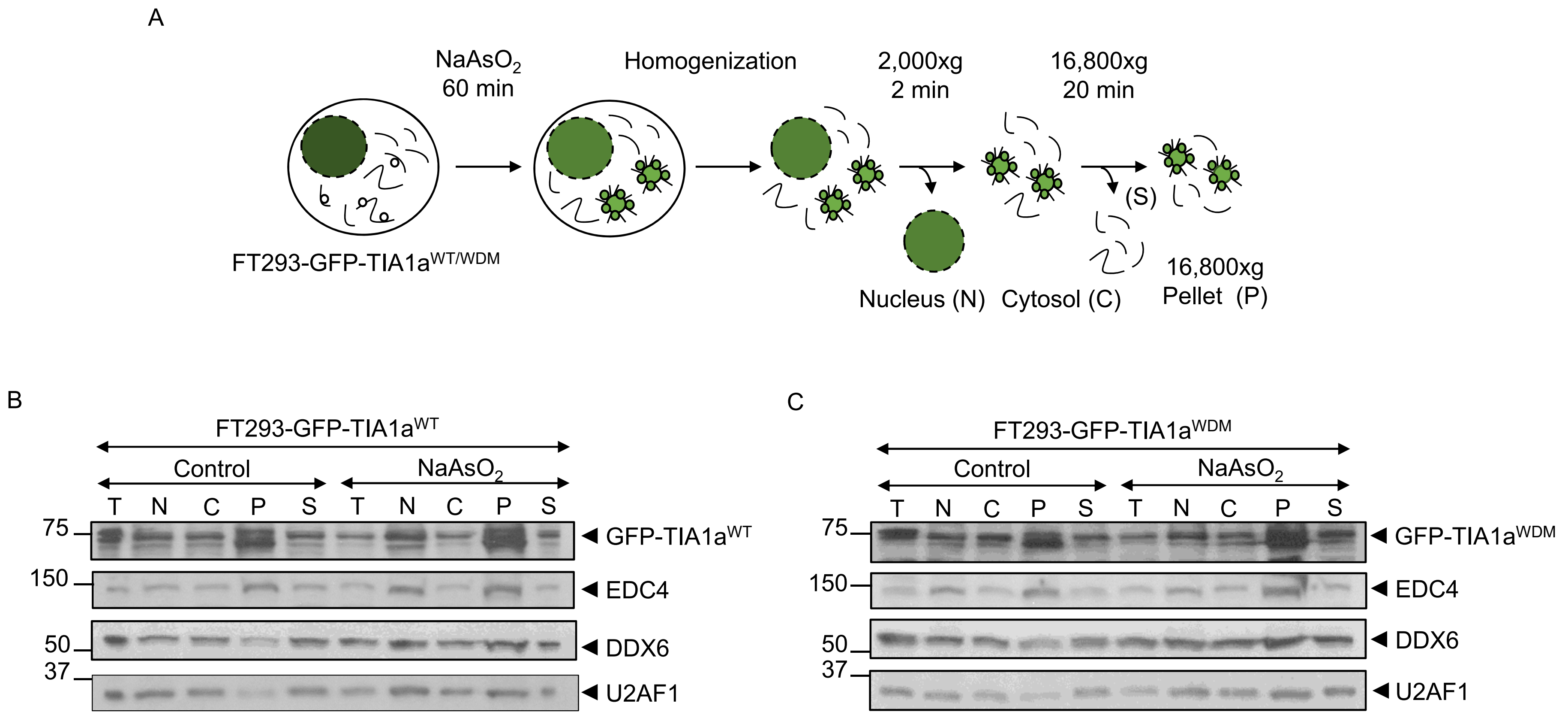
A
NaAsO2
60 min
Homogenization
2,000xg
2 min
16,800xg
20 min
FT293-GFP-TIA1a WT/WDM
(S)
16,800xg
Pellet (P)
Nucleus (N)
Cytosol (C)
B
FT293-GFP-TIA1a WT
Control
NaAsO2
T N C P S T N C P S
75
150
50
37
GFP-TIA1a WT
EDC4
DDX6
U2AF1
C
FT293-GFP-TIA1a WDM
Control
NaAsO2
T N C P S T N C P S
75
150
50
37
GFP-TIA1a WDM
EDC4
DDX6
U2AF1

Figure S11

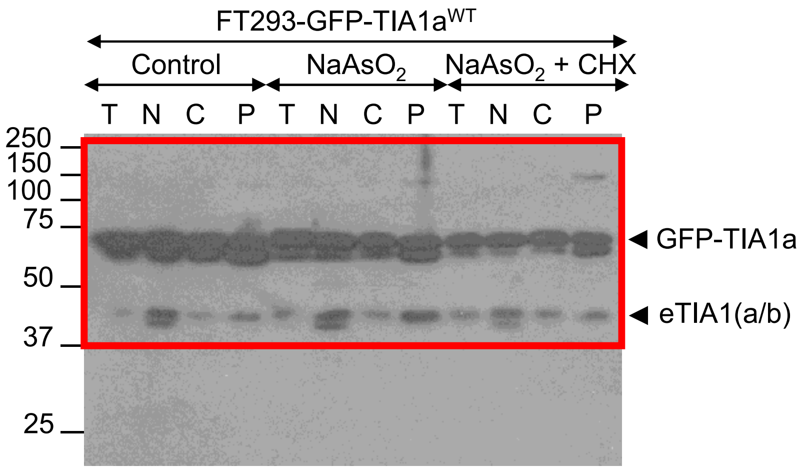

Upper Western blot of Figure S1B

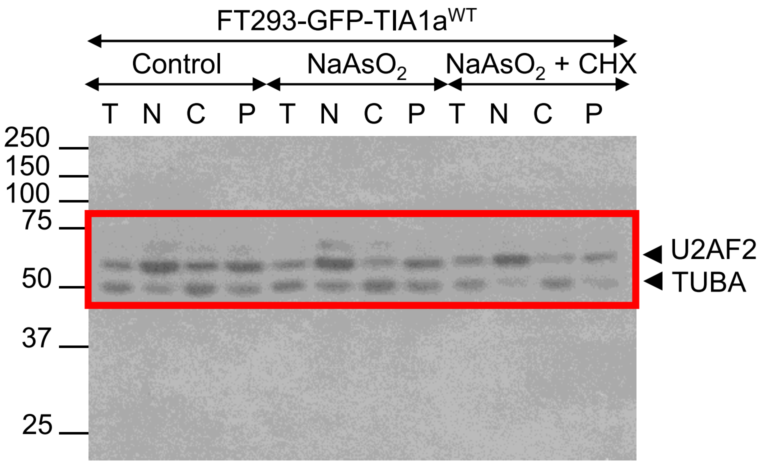

Lower Western blot of Figure S1B

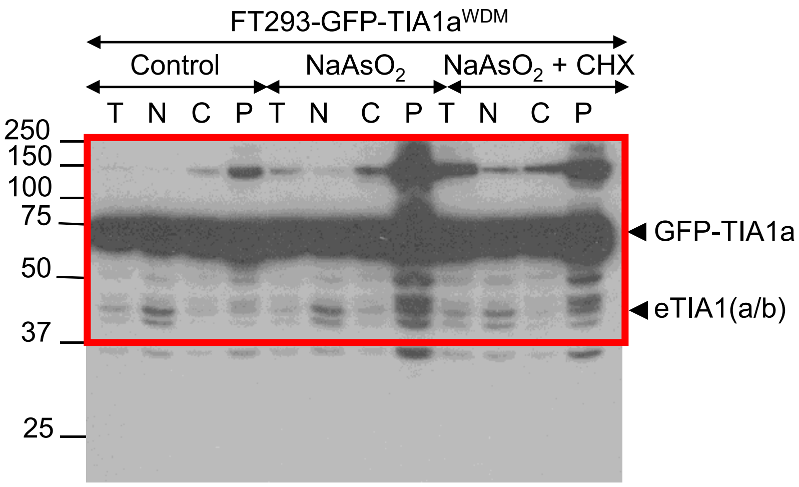

Upper Western blot of Figure S1C

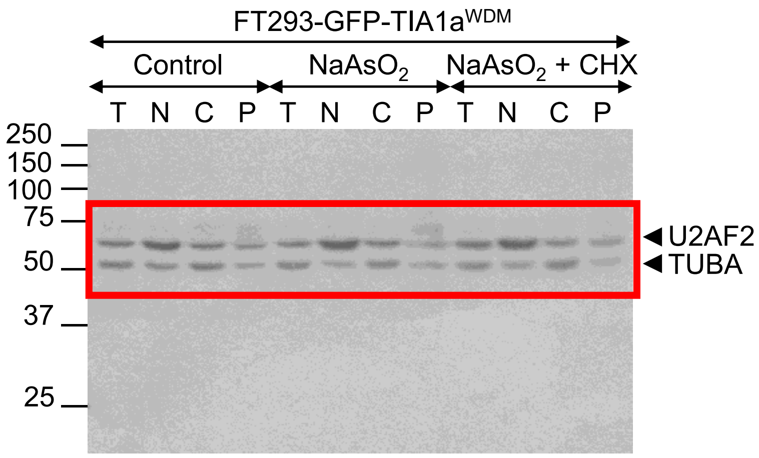

Lower Western blot of Figure S1C

Figure S12

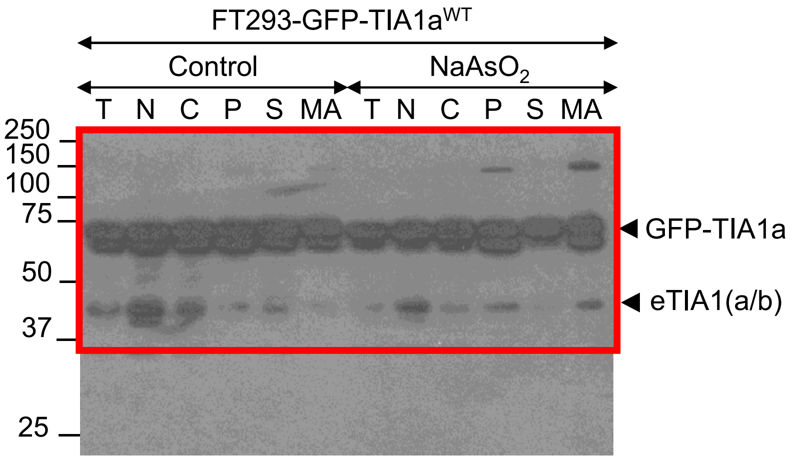

Upper Western blot of Figure S1D

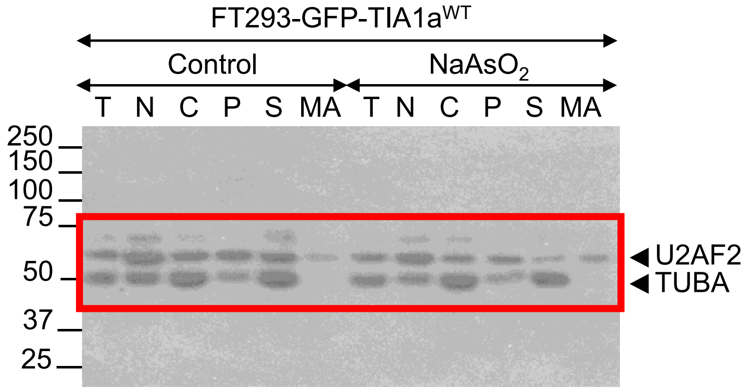

Lower Western blot of Figure S1D

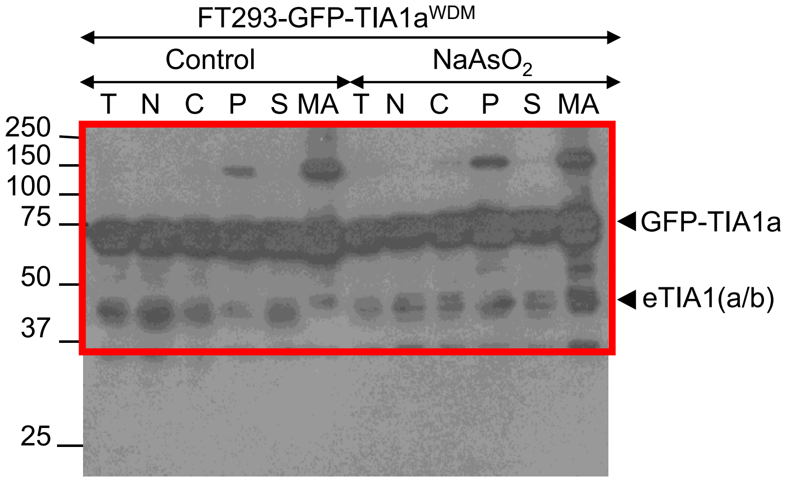

Upper Western blot of Figure S1E

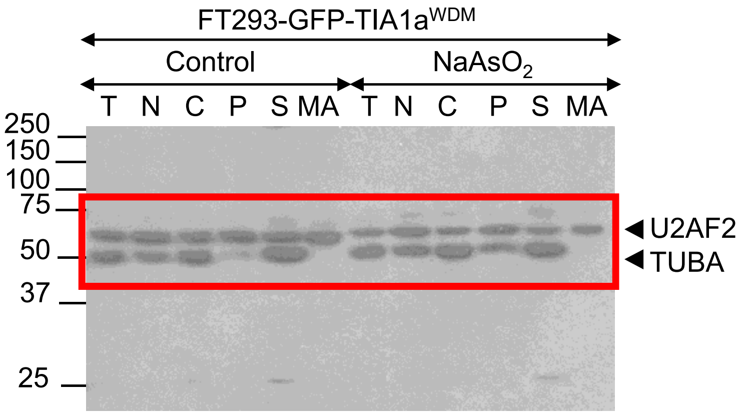

Lower Western blot of Figure S1E

Figure S13

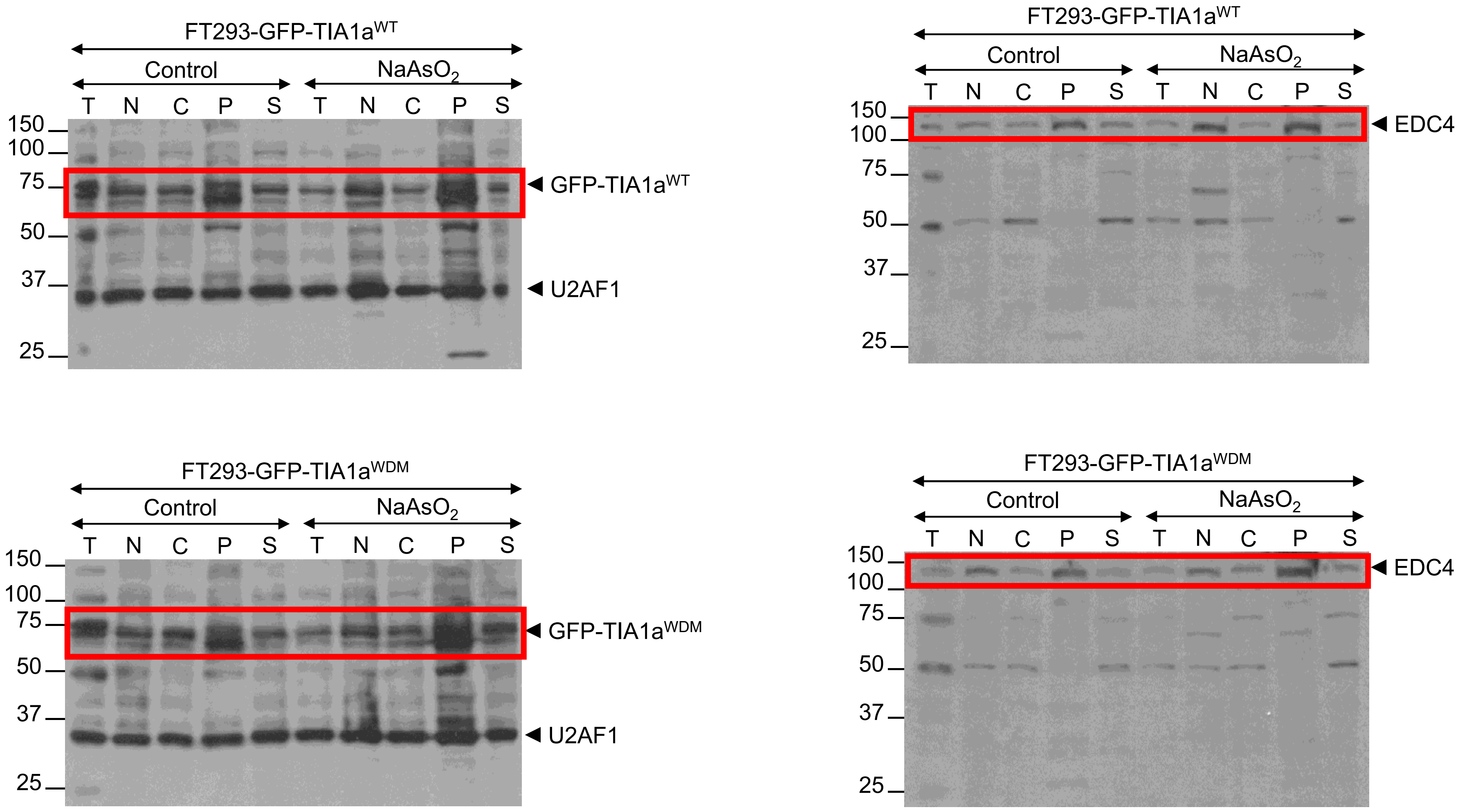

Western blots of Figure S10

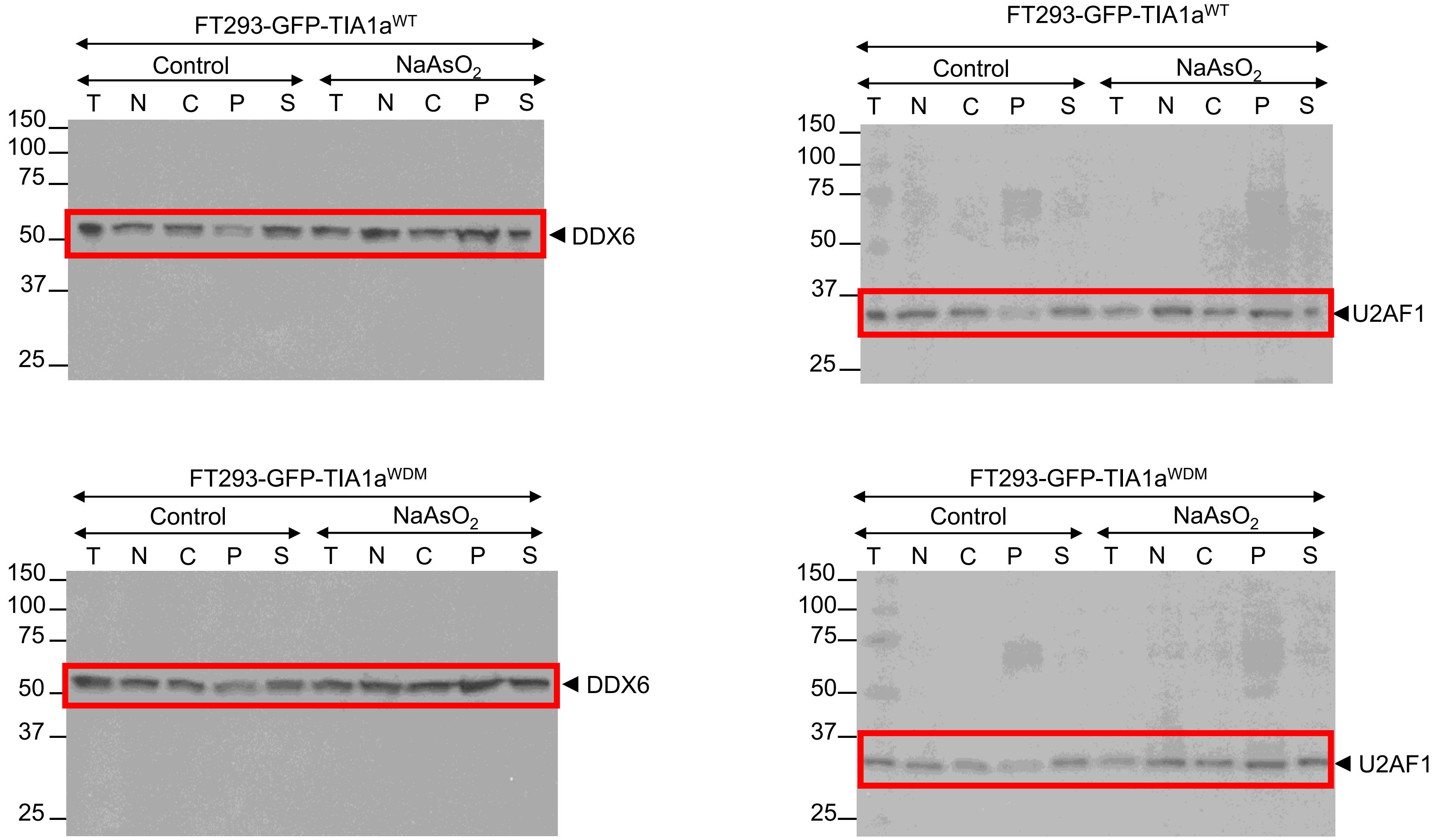

Western blots of Figure S10
